# An Integrated Morphological and Molecular Approach to the Description and Systematisation of a Novel Genus and Species of Macrodasyida (Gastrotricha)

**DOI:** 10.1371/journal.pone.0130278

**Published:** 2015-07-08

**Authors:** M. Antonio Todaro, Matteo Dal Zotto, Francesca Leasi

**Affiliations:** 1 Department of Life Sciences, University of Modena and Reggio Emilia, Modena, Italy; 2 Consorzio Interuniversitario per il Centro di Biologia Marina ed Ecologia Applicata ‘G. Bacci’, Livorno, Italy; 3 National Museum of Natural History, Smithsonian Institution, Washington, D.C., United States of America; Virginia Commonwealth Univ, UNITED STATES

## Abstract

**Background:**

Gastrotricha systematics is in a state of flux mainly due to the conflicts between cladistic studies base on molecular markers and the classical systematisation based on morphological traits. In sandy samples from Thailand, we found numerous macrodasyidan gastrotrichs belonging to an undescribed species of difficult taxonomic affiliation. The abundance and original nature of the specimens prompted us to undertake a deep survey of both morphological and molecular traits aiming at a reliable systematisation of the new taxon.

**Methodology/Principal Findings:**

Using several microscopical techniques we investigated the external and internal anatomy, including the muscular and nervous systems of the new species. Additional specimens were used to obtain the 18S rRNA gene sequence; molecular data was analysed cladistically in conjunction with data from additional species belonging to the near complete Macrodasyida taxonomic spectrum. Specimens are vermiform, up to 806 μm in total length, and show a well-defined head equipped with peculiar leaf-like sensorial organs and a single-lobed posterior end. The adhesive apparatus includes anterior, ventrolateral, dorsal and posterior tubes. Pharynx is about 1/4 of the total length and shows pores at its posterior 3/4. Adult specimens exhibit maturing eggs and a bulky, muscular caudal organ, but do not show sperm nor the frontal organ. Musculature and nervous system organisation resemble the usual macrodasyidan plan; however, the somatic circular muscles of the intestinal region surround all other muscular components and a third FMRFamide-IR commissure ventral to the pharyngo-intestinal junction appear to be an autoapomorphic traits of the new species.

**Conclusions/Significance:**

While the anatomical characteristics of the Asian specimens appear so unique to grant the establishment of a new taxon, for which the name Thaidasys tongiorgii gen. et sp. nov. is proposed, the result of phylogenetic analyses based on the 18S rRNA gene unites the new genus with the family Macrodasyidae.

## Introduction

Gastrotricha is a phylum of aquatic microinvertebrates, which contains about 820 accepted species (as of April 2015) divided into two orders: Chaetonotida that includes tenpin-shaped, hermaphroditic and/or parthenogenetic species found in marine, brackish or freshwater habitats, and Macrodasyida, a group of vermiform, hermaphroditic species that live interstitially, mostly in marine sand (e.g., [1, 2]). Knowledge on the alpha biodiversity of the entire phylum is growing at a fast pace due to the continual description of new species (e.g., freshwater: [3–7]; marine: [8–22]), whereas recent cladistics studies challenging the phylogenetic congruence of the classical systematisation have notably increased the number of recognised genera and families [21, 23–26]. Currently, the order Chaetonotida is subdivided into 8 families and 31 genera, while the order Macrodasyida includes 10 families and 34 genera (e.g., [27]). However, the effort to make systematisation more congruent with the results of phylogenetic studies is far from completed, as best testified by the recent work on the largest family of the phylum [28].

One of the problems that makes revision difficult is that the anatomical ground patterns of taxa putatively belonging to the different evolutionary lines are still not well-known (e.g., [1, 29–32]). Since the process of re-systematisation benefits from additional surveys of insufficiently known taxa [33], the discovery of new species with novel characteristics could help to identify plesiomorphy in these morphologically diverse animals, thus providing a more solid ground for their natural grouping [1, 21].

Herein, a new interesting macrodasyidan species sampled from a beach at Phuket Island, Thailand is described. Using several microscopical techniques, namely Differential Interference Contrast (DIC), Scanning Electron (SEM) and Confocal Laser Scanning Microscopy (CLSM), we investigated the external and internal anatomy including the muscular and nervous systems. While the external morphology and traits of the reproductive system of the new species appear so unique among Gastrotricha to grant the creation of a new genus, reliable clues about its phylogenetic alliances at a higher taxonomic (family) levels only came from cladistic analyses based on the 18S rRNA gene that included all of the relevant taxa of the order Macrodasyida.

## Material and Methods

### Sampling

Sampling took place in February 2010 at Kata beach (Phuket island, Thailand); during high tide about 500 ml of sand was collected by hand at a depth of 0.5 m using a plastic jar [34]; thereafter, sand was kept refrigerated in an insulated bag and brought to the laboratory in Modena, Italy within 48 hrs. No special permission/permits were needed to collect these animals as gastrotrichs are microscopic, non-pathogenic organisms; field studies did not involve endangered species and sampling was carried out on a public beach.

### Morphological analysis

#### Gastrotrich extraction and Differential Interference Contrast microscopy

In the laboratory, the specimens were extracted daily with the narcotisation-decantation technique using a 7% magnesium chloride solution within one week of collection; the supernatant was poured into plastic Petri dishes (3 cm diameter) and scanned for gastrotrichs at a maximum magnification of 50 x under a Wild M8 stereomicroscope [35]. When located, each individual gastrotrich specimen was mounted on a glass slide and observed *in vivo* with Nomarski differential interference contrast optics using a Nikon Eclipse 90i microscope. During observation, eight specimens were photographed with a DS-5M Nikon digital camera and measured using the Nikon ACT-2U software v.1.4. Additional specimens were identified and prepared for scanning electron- or confocal microscopy while three more were fixed in 95% ethanol and stored for DNA analysis.

#### Scanning Electron Microscopy

Four formalin-fixed speciemens were rinsed in in 0.1 M PBS buffer, dehydrated through a graded ethanol series, critical point-dried using CO_2_, mounted on aluminium stubs, sputter-coated with gold-palladium, and observed with a Philips XL 30 scanning electron microscope [36].

The description of the new species follows the scheme adopted by Hummon *et al*. [37], where the locations of some morphological characteristics along the body are given in percentage units (U) of total body length measured from the anterior to posterior.

#### Confocal Laser Scanning Microscopy

Up to fifteen mature, relaxed specimens were incubated at 4°C for 1 hour in 4% formaldehyde solution (freshly made from paraformaldehyde in 0.1M Phosphate Buffered Saline (PBS); pH 7.4) and subsequently prepared for survey of the muscle and nervous systems. For musculature observations, five fixed specimens were washed several times with 0.1M PBS, permeabilised for 1hr in a PBT preincubation solution (0.2% Triton X-100, 0.25% Bovine Serum Albumin (BSA), and 0.05% NaN_3_ in PBS 0.1M), incubated in TRITC-phalloidin (Sigma) (8 μl 38 μM solution in 200 μl preincubation solution) for 1 hour, rinsed again in PBS and embedded in 3% DABCO (Sigma, Italy) on microscope slides [38]. For surveys of the nervous system, ten fixed specimens were blocked for 2–3 h with normal goat serum (50% in PBS) at room temperature. The serum was previously deactivated at 55°C for 30 min. The specimens were then transferred to a rabbit polyclonal serotonin (5-HT) antibody or a rabbit polyclonal FMRFamide antibody together with a goat polyclonal α-tubulin antibody (Sigma-Aldrich, 1:200 PBT), and kept on an orbital shaker overnight at 4°C. After being rinsed in 0.1 M PBS, the animals were transferred to BSA (1% in) PBS and then into the secondary antibody (anti-rabbit, Alexa Fluor 488 for serotonin and FMRFamide; anti-goat Alexa Fluor 633 for x-tubulin; 1:500 in PBT) overnight at 4°C. Samples were then rinsed in 0.1 M PBT. Some of the processed animals were stained with DAPI (4’,6-diamidino-2-phenylindole; Sigma, Italy). Two additional control groups of two specimens were each processed to assess the specificity of the immune-cytochemical response; one control group had the primary antibody omitted and another was incubated with preabsorbed antibody; finally, specimens were mounted in 3% DABCO (Sigma, Italy) [39]. Observation of both the muscular and nervous systems was performed using a Leica DM IRE 2 Confocal Laser Scanning Microscope. A series of optical sections were projected in one maximum-projection (MPJ) image, or visualized as a simulated fluorescence projection (SFPJ) for three-dimensional appearance.

### Granulometry and Abundance

Granulometric analysis of the substrata was carried out according to Todaro *et al*. [40]. Mean grain size, sorting coefficient, kurtosis, and skewness were calculated by a computerised programme based on the equation of Seward-Thompson & Hails [41]. The rationale for abundance of the species among other species of a sample is as follows: rare, less than 1% of a sample; scarce, 3–5% of a sample; numerous, 10–20% of a sample (often a sub-dominant); and prevalent, more than 30% of a sample (usually dominant or co-dominant) [42].

### Molecular analysis

To estimate the phylogenetic relationships of the new taxon within the order Macrodasyida, the near complete 18S rRNA sequence of 44 species (45 specimens) belonging to 24 genera within the ten currently recognised families were used (Table 1). A representative of the order Chaetonotida, *Xenotrichula intermedia* (Xenotrichulidae), was chosen as the out-group in the analyses. The sequences used are the same as in Todaro *et al*. [21].

**Table 1:** Gastrotrich taxa involved in the molecular analyses. Origin, reference and GenBank accession number are provided.

Taxon	Origin	Reference	Accession
**Cephalodasyidae**			
*Cephalodasys* sp	White Sea, Russia	[43]	AY963691
*Dolichodasys* sp.	San Isidoro, Italy	[44]	AM231778
*Mesodasys laticaudatus*	Albinia, Italy	[45]	JF357657
*Mesodasys littoralis*	Bou Ficha, Tunisia	[45]	JF357658
*Paradasys* sp.	Ionian sea, Italy	[44]	AM231781
*Pleurodasys helgolandicus*	Ibiza, Spain	[24]	JN203486
**Dactylopodolidae**			
*Dactylopodola* cf. *baltica*	Ras Alard, Kuwait	[45]	JF357650
*Dactylopodola mesotyphle*	Punta Ala, Italy	[45]	JF357651
*Dactylopodola typhle*	Bou Ficha, Tunisia	[45]	JF357652
*Dactylopodola typhle*	Torre Civette, Italy	[45]	JF357653
**Hummondasyidae**			
*Hummondasys jamaicensis*	Negril, Jamaica	[21]	KM083602
**Lepidodasyidae**			
*Lepidodasys unicarenatus*	Pianosa, Italy	[45]	JF357665
**Macrodasyidae**			
*Macrodasys* sp. 1	Torre Civette, Italy	[45]	JF357654
*Macrodasys* sp. 2	Bohuslän, Sweden	[45]	JF357670
*Thaidasys tongiorgii*	Phuket island, Thailand	Present study	KR072683
*Urodasys* sp.	NA	[46]	AY218102
*Urodasys* sp.1	Florida, USA	[47]	DQ079912
**Planodasyidae**			
*Crasiella* sp.	Ilha Bela, Brazil	[24]	JN203488
*Megadasys* sp.	Grotta del Ciolo, Italy	[45]	JF357655
*Megadasys* sp. 1	Porto Cesareo, Italy	[45]	JF357656
**Redudasyidae**			
*Anandrodasys agadasys*	St. John Island, USA	[24]	JN203487
*Redudasys fornerise*	Represa do Broa, Brazil	[24]	JN203489
**Thumastodermatidae**			
*Acanthodasys* sp. a	Capraia, Italy	[45]	JF357638
*Acanthodasys aculeatus*	Capraia, Italy	[45]	JF357639
*Diplodasys ankeli*	Meloria, Italy	[45]	JF357624
*Diplodasys meloriae*	Meloria, Italy	[45]	JF357640
*Oregodasys ocellatus*	Meloria, Italy	[45]	JF357642
*Oregodasys ruber*	Meloria, Italy	[45]	JF357625
*Oregodasys tentaculatus*	Meloria, Italy	[45]	JF357626
*Pseudostomella etrusca*	Albinia, Italy	[45]	JF357633
*Ptychostomella lamelliphora* (= sp1)	Ilha Bela, Brazil	[45]	JF357643
*Ptychostomella tyrrhenica*	Albinia, Italy	[45]	JF357634
*Tetranchyroderma papii*	Sardegna, Italy	[45]	JF357637
*Tetranchyroderma esarabdophorum*	Mahdia, Tunisia	[45]	JF357627
*Tetranchyroderma hirtum*	Capraia, Italy	[45]	JF357628
*Tetranchyroderma thysanophorum*	Albinia, Italy	[45]	JF357630
*Thaumastoderma moebjergi*	Bohuslxn, Sweden	[45]	JF357671
*Thaumastoderma ramuliferum*	Meloria, Italy	[45]	JF357631
**Turbanellidae**			
*Paraturbanella dohrni*	Punta Ala, Italy	[45]	JF357659
*Paraturbanella pallida*	Capraia, Italy	[45]	JF357660
*Paraturbanella teissieri*	Punta Ala, Italy	[45]	JF357661
*Turbanella bocqueti*	Tramore, Ireland	[45]	JF357662
*Turbanella cornuta*	Chioggia, Italy	[45]	JF357663
*Turbanella lutheri*	Torö, Sweden	[45]	JF357669
**Xenodasyidae**			
*Xenodasys riedli*	St. John Island, USA	[24]	JN203490
Xenotrichulidae*			
*Xenotrichula intermedia*	Mahdia, Tunisia	[45]	JF357664

* Order Chaetonotida; NA, Data not available.

With regard to the new taxon, DNA was extracted from a single whole specimen using the QIAamp DNA mini kit (QIAGEN), with columns from the QIAamp DNA micro kit (QIAGEN), according to the manufacturer’s instructions. The extract was then used as a template for the subsequent amplifications. A 1712 bp fragment of DNA was amplified using the 0.2 ml PuReTaq Ready-To-Go PCR beads (GE Healthcare). For amplification, 0.5 ml of each primer, 2 ml of DNA and 22 ml of purified water were assembled in the RTG-PCR tubes, yielding a final volume of 25 ml. Primer sequences and PCR-programs are from Todaro *et al*. [45], with the polymerase chain reactions carried out in a Biometra personal thermocycler. The PCR-products were purified using the QIAquick PCR Purification Kit (QIAGEN) according to the manufacturer’s instructions and sent for sequencing to Macrogen, Korea (www.macrogen.co.kr). Contigs were assembled using Staden v1.6.0 [48]. The 45 sequences were aligned with MUSCLE (Multiple sequence comparison by Log-Expectation), as implemented in MEGA 6 [49], using the default parameters. The data set, which consisted of 1895 nucleotide characters, was subsequently converted into both interleaved Nexus and Fasta formatted files and analysed phylogenetically using three different approaches: 1) Maximum Parsimony (MP, MEGA 6), 2) Maximum Likelihood (ML, MEGA 6) and 3), Bayesian inference (BI, MrBayes 3.1.2) [50]. For the analyses carried out with ML and BI the evolutionary model of nucleotide substitution GTR+G+I was used, which is favoured by both the AICc and the lnL criteria in MrModeltest v2.3 [51] and Mega 6. For both the ML and MP analyses, the ‘‘use-all sites” data treatment option was selected, and node support was generated using 1000 bootstrap replicates. For the Bayesian analysis, two independent runs, each with four simultaneous chains were run for 6,000,000 generations; trees were sampled every 100th generation, and posterior probabilities were determined after a burn-in of 15000 generations. A 50% consensus tree was produced with TreeView [52].

### Nomenclatural acts

The electronic edition of this article conforms to the requirements of the amended International Code of Zoological Nomenclature, and hence the new names contained herein are available under that Code from the electronic edition of this article. This published work and the nomenclatural acts it contains have been registered in ZooBank, the online registration system for the ICZN. The ZooBank LSIDs (Life Science Identifiers) can be resolved and the associated information viewed through any standard Web browser by appending the LSID to the prefix "http://zoobank.org/". The LSID for this publication is: urn:lsid:zoobank.org:pub:D2086FAC-1B6F-4DA4-B080-702A05BE9EBE. The electronic edition of this work was published in a journal with an ISSN, and has been archived and is available from the following digital repositories: PubMed Central, LOCKSS.

## Results

### Taxonomic treatment

Order Macrodasyida Remane, 1925 [Rao & Clausen, 1970]

Family Macrodasyidae Remane, 1924

Genus *Thaidasys* gen. nov.

urn:lsid:zoobank.org:act:4920EC03-B601-4C6E-80B3-C90354097FCA

#### Diagnosis

Body elongate, up to 806 μm in total length (LT), and rather narrow, up to 68 μm in width, flattened ventrally and vaulted dorsally, with numerous epidermal glands. Cuticular covering smooth, devoid of scales and/or spines. Head consisting of a well demarcated anterior region showing a peculiar leaf-like structure on each posterior side, and a posterior region, hosting the brain, comprised between two evident constrictions. Posterior body region unilobed, ovoidal in shape. Sensory hairs arranged singly in lateral and dorsolateral columns along the body, sparsely on the lateral sides of the head but forming a dense, semi-circular fringe on the head. Ventral locomotor ciliature in the form of two bands running separately from under the head to the posterior trunk region; cilia in the bands appear rather sparse especially in the rear end. Anterior adhesive tubes (TbA), 2–3 per side, forming diagonal columns, which insert directly on the body surface and project forward; ventral adhesive tubes (TbV), absent; ventrolateral adhesive tubes (TbVL), up to 24 per side; five-six along the pharyngeal region and the remaining along the intestinal region; dorsal adhesive tubes (TbD), up to 15 per side, three of which are present on the posterior half of the pharyngeal region; dorsolateral adhesive tubes (TbDL), absent; posterior adhesive tubes (TbP), up to 6, surrounding the caudum. Mouth terminal, of mid-size (up to 15 μm in diameter), leading to a short buccal cavity (8–10 xm in length), which opens into a 192–209 μm long and 15–22 μm wide pharynx; pharyngeal pores far-off from the base with ventrolateral openings. Pharyngo-intestinal junction (PhIJ) at about U26. Intestine increases in width from the PhIJ to mid-body and gradually narrows toward the posterior body end; anus ventral at U96. Testes and/or sperm absent/not seen. Female gonad unpaired, in the second third of the trunk, showing oocytes maturing in a caudo-cephalic direction with largest egg dorsal to the mid intestine. A noticeable muscular organ in the posterior trunk region (U83), following the ovary is present. The structure is ventral to the intestine, tube-like, up to 76 μm in length and 24 μm in width, with a strongly muscularised wall and a throughout canal, 6–8 μm in diameter; it is interpreted as the caudal organ.

#### Etymology

The genus is named after the country where the these animals were first found

*Thaidasys tongiorgii* sp. nov.

urn:lsid:zoobank.org:act:26CF6C3E-5975-450F-82A5-E7333E99732B

(Figs 1–11)

**Fig 1:**
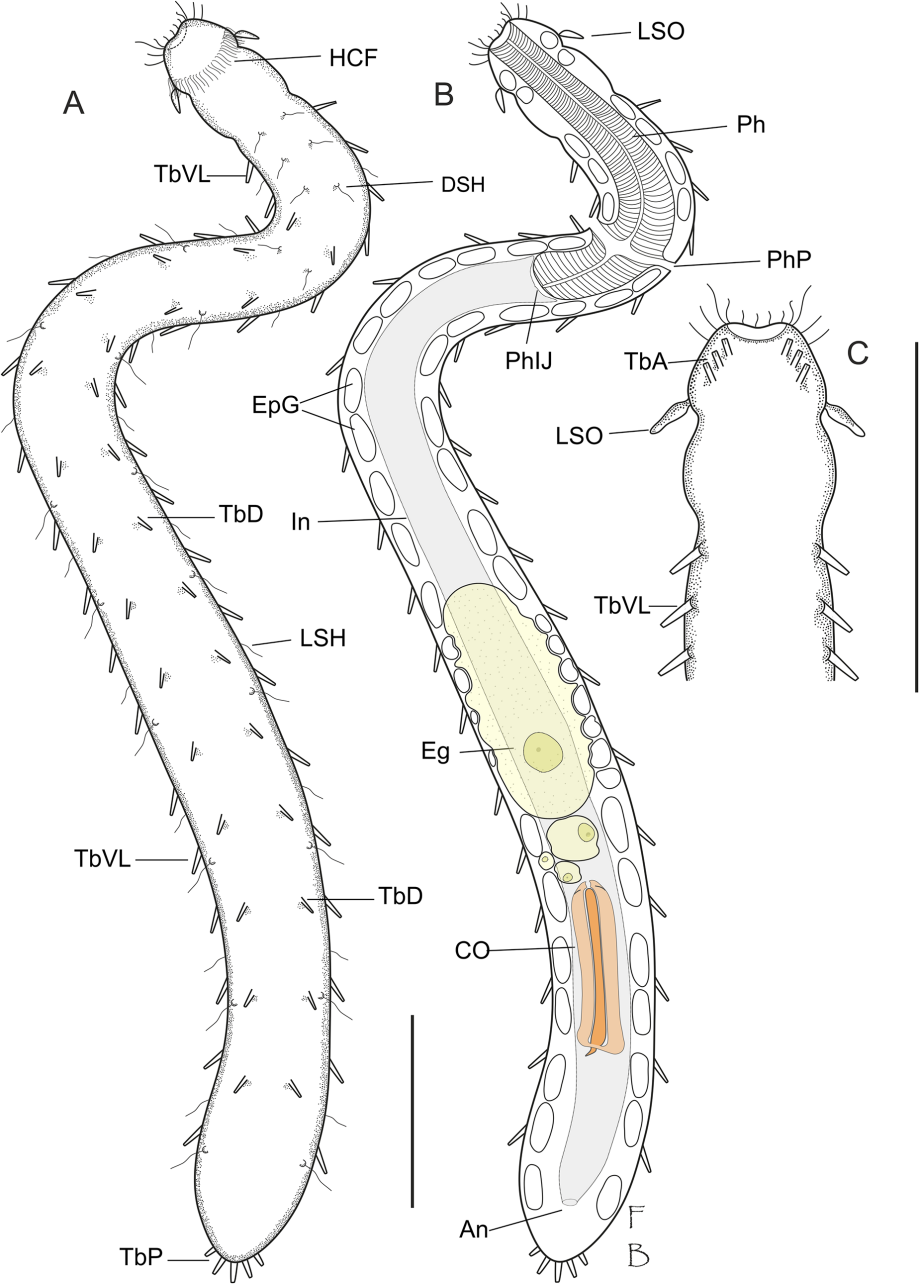
Line art illustrations of *Thaidasys tongiorgii* gen. et sp. nov. A, habitus as seen from the dorsal side; B, same as the previous but at a different focal plane, showing the internal anatomy with the maturing oocytes and the putative caudal organ; C, anterior region seen from the ventral side, showing the arrangement of the anterior adhesive tubes. An, anus; CO, caudal organ; DSH, dorsal sensory hair; Eg, egg; EpG, epidermal gland; In, intestine; HCF, head ciliary fringe; LSH, lateral sensory hair; LSO, leaf-like sensorial organ; Ph, pharynx; PhIJ, pharyngo-intestinal junction; PhP, pharyngeal pore; TbA, anterior adhesive tube; TbD, dorsal adhesive tube; TbP, posterior adhesive tube; TbVL, ventrolateral adhesive tube. Drawings are made mostly from the specimen shown in Fig 2. Scale bars = 100 μm.

**Fig 2:**
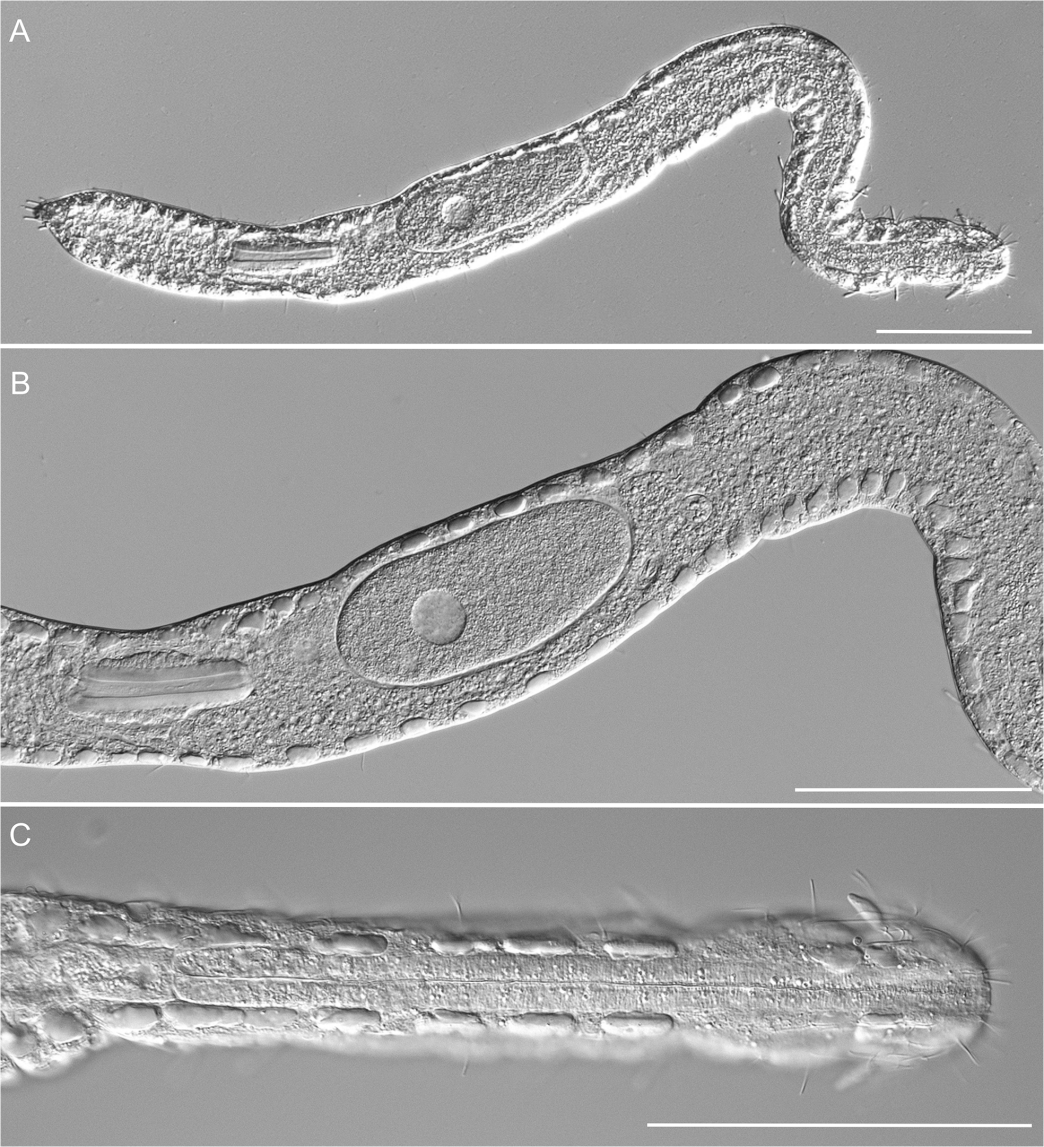
*Thaidasys tongiorgii* gen. et sp. nov. Differential interference contrast photomicrographs. A, habitus of the holotype; B, close-up of the trunk, showing the maturing eggs and the putative caudal organ; C, close-up of the anterior region of a different specimen, showing the head with the leaf-like sensorial organs, the pharynx and the epidermal glands. Scale bars = 100 μm.

**Fig 3:**
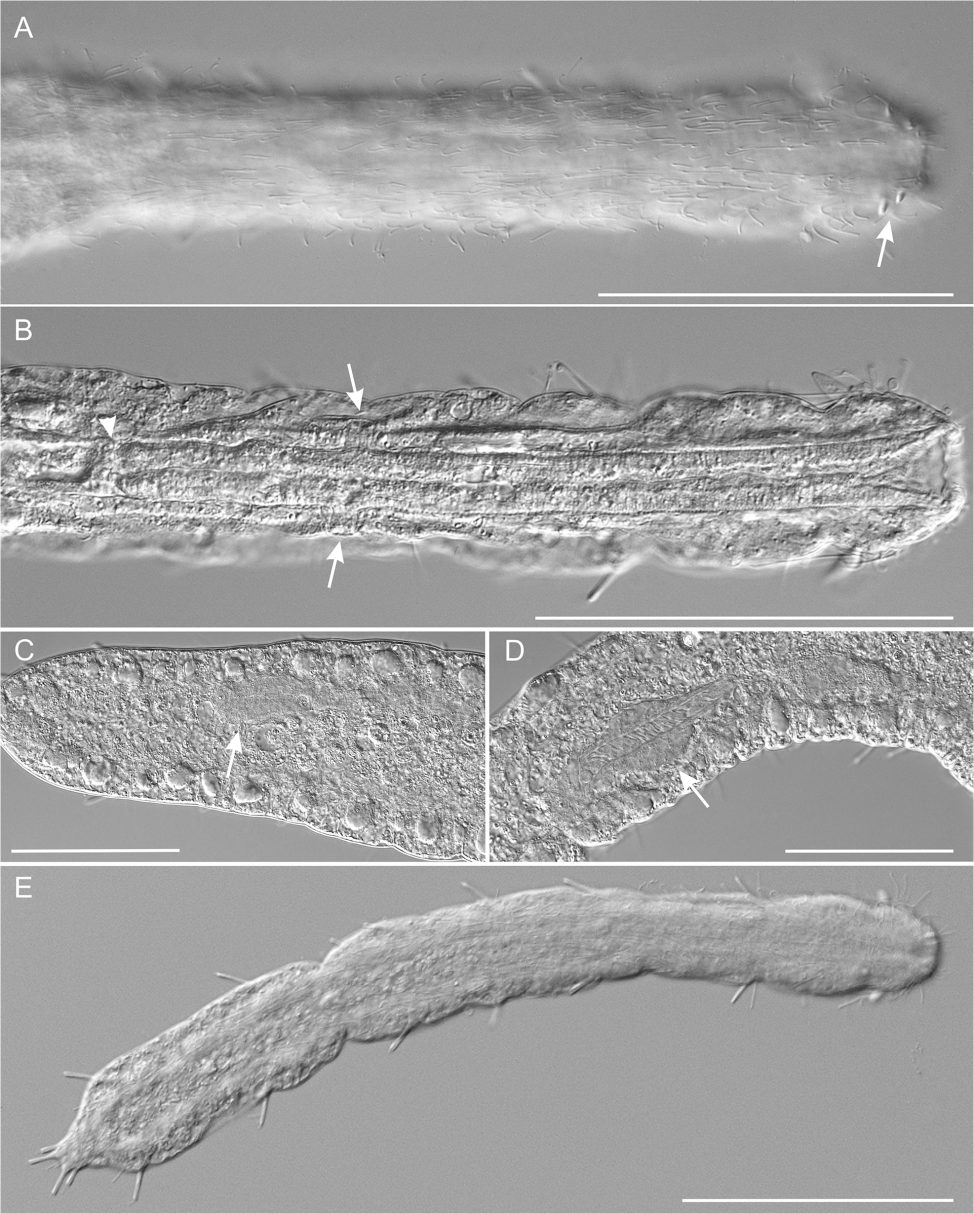
*Thaidasys tongiorgii* gen. et sp. nov. Differential interference contrast photomicrographs. A, close-up of the anterior region, ventral view, showing the locomotor ciliation and the anterior adhesive tubes (arrow); B, close-up of the internal view of the anterior region of a different specimen, showing the pharynx, pharyngeal pores (arrows) and pharyngo-intestinal junction (arrowhead). C, D, Posterior region of the trunk of two subadult specimens, showing the caudal organ (arrows) at different development stages. E, a juvenile. Scale bars A, B, E = 100 μm, C, D = 50 μm.

**Fig 4:**
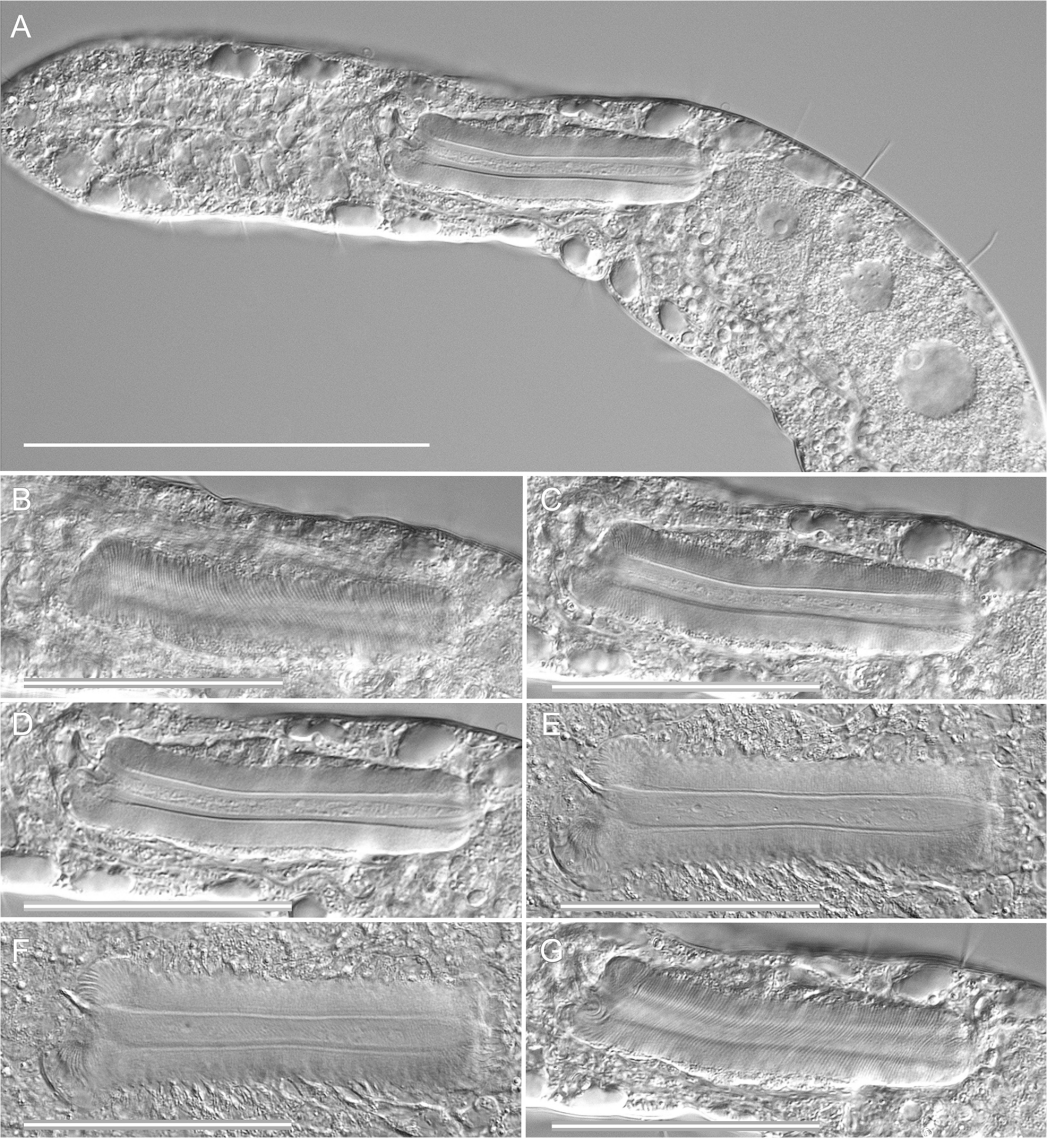
*Thaidasys tongiorgii* gen. et sp. nov. Differential interference contrast photomicrographs. A, close-up of the trunk region, internal view, showing the maturing oocytes followed by the putative caudal organ; B-G, caudal organ at different focal planes, from dorsal to ventral. Scale bars A = 100 μm, B-G = 50 μm.

**Fig 5:**
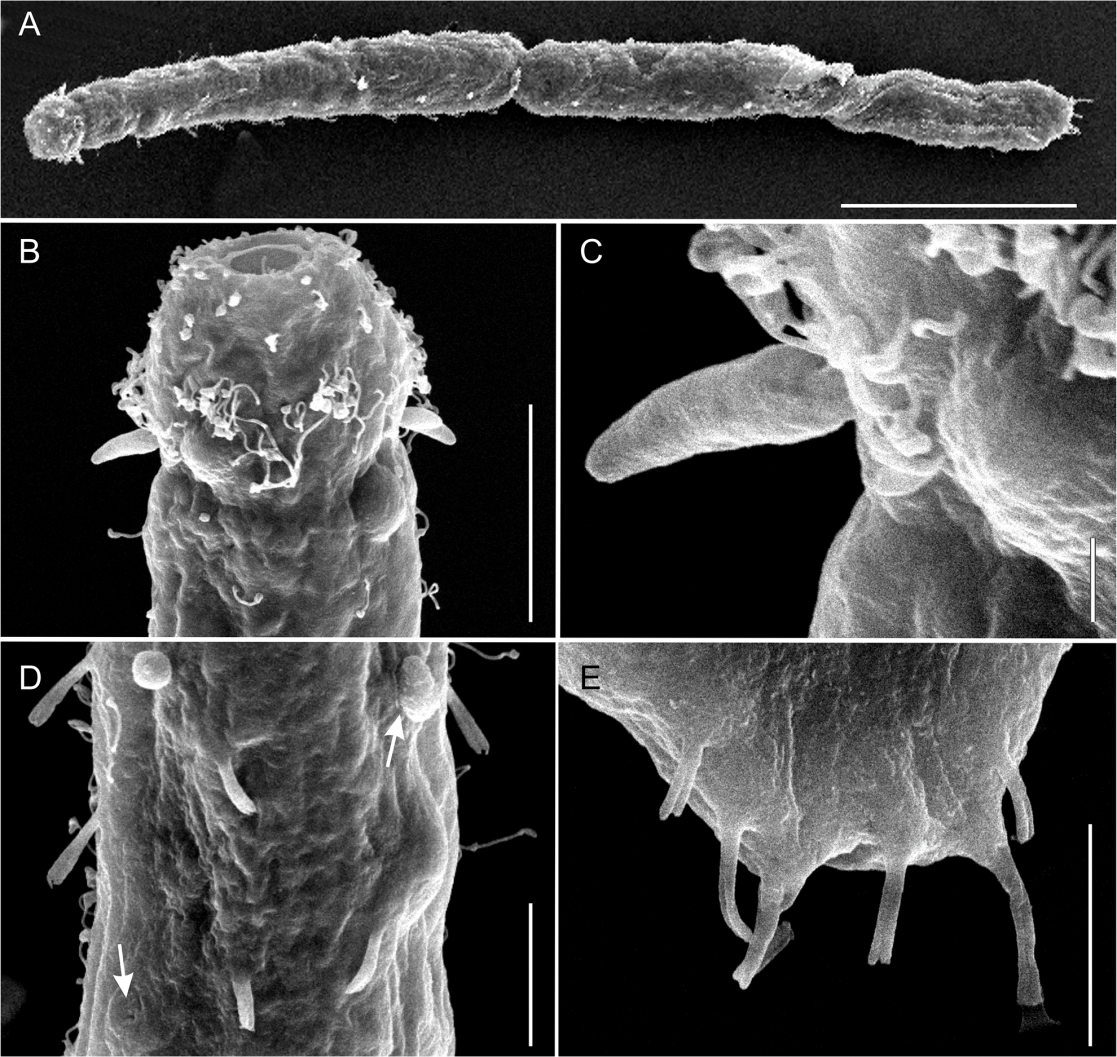
*Thaidasys tongiorgii* gen. et sp. nov. Scanning electron microscope photomicrographs. A, habitus, mostly dorsal view, but with a twisted posterior end; B, anterior end, showing the head and the mouth; C, close-up of the head leaf-like sensorial organ; D, close up pharyngeal region, showing the dorsal tubes and the opening of the epidermal glands (arrows); E, close up of the caudum, ventral view, showing the arrangement of the posterior adhesive tubes. Scale bars: A = 100 μm, B = 20 μm, C = 2 μm, D, E = 10 μm.

**Fig 6:**
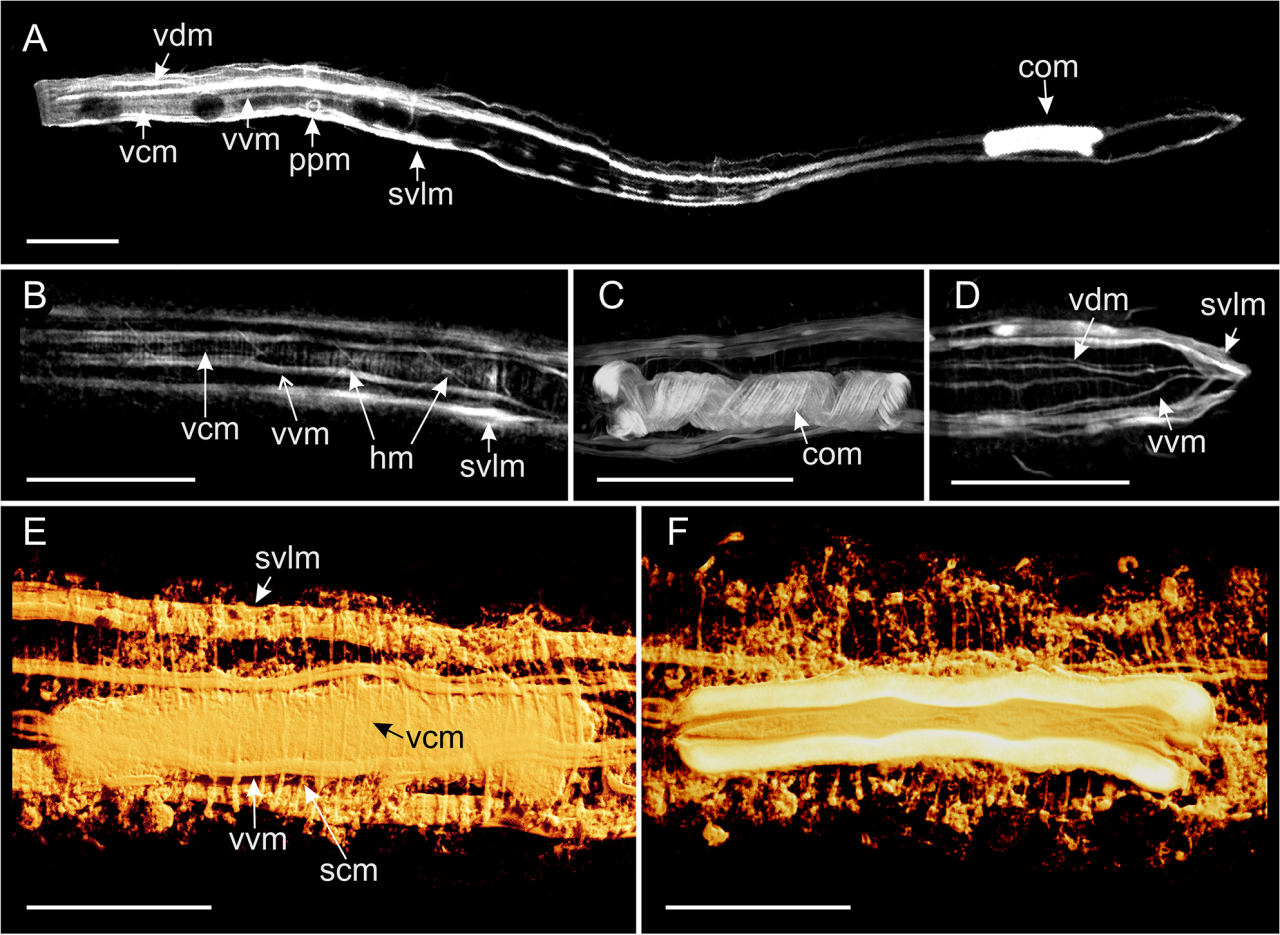
*Thaidasys tongiorgii* gen. et sp. nov. Muscular system visualised with CLSM. A, whole organism. B, close up of the pharynx, showing circular, helicoidal and longitudinal muscles. C, musculature supplying the caudal organ. D, posterior end. E, F, three-dimensional reconstructions of the musculature of the complete (E) and internal (F) view of the caudal organ. com, musculature supplying the caudal organ; hm, helicoidal muscles; ppm, muscular ring supplying the pharyngeal pore; scm, somatic circular muscles; svlm, somatic ventrolateral muscles; vcm, visceral circular muscles; vdm, visceral dorsal longitudinal muscles; vvm, visceral ventral longitudinal muscles. Scale bars A-D = 50 μm, E, F = 25 μm.

**Fig 7:**
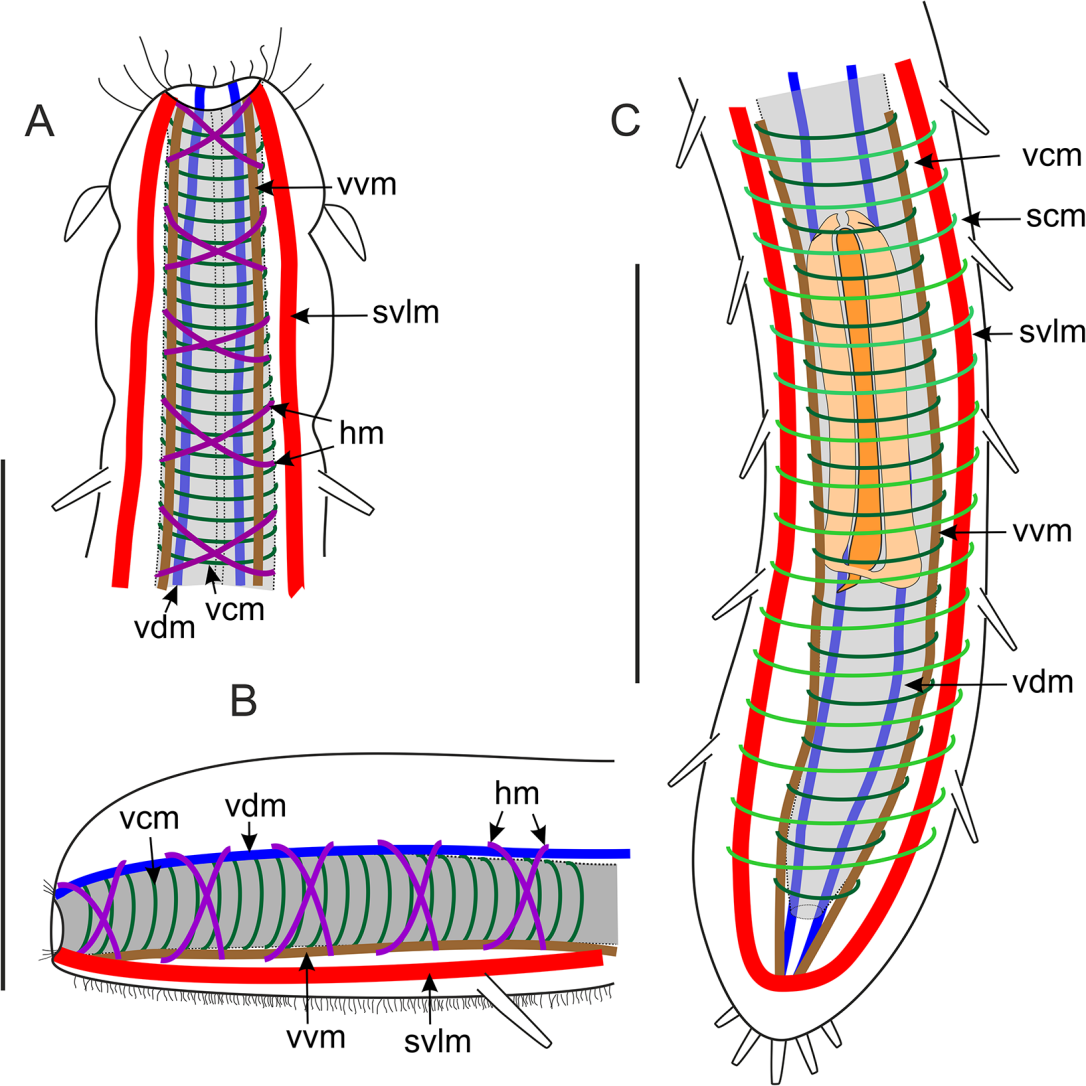
*Thaidasys tongiorgii* gen. et sp. nov. Schematic drawings of the muscular system. A, Anterior region, ventral view. B, Anterior region, lateral view. C, Posterior region, ventral view. hm, helicoidal muscles; scm, somatic circular muscles; svlm, somatic ventrolateral muscles; vcm, visceral circular muscles; vdm, visceral dorsal longitudinal muscles; vvm, visceral ventral longitudinal muscles. Scale bars 100 μm (A, B same scale).

**Fig 8:**
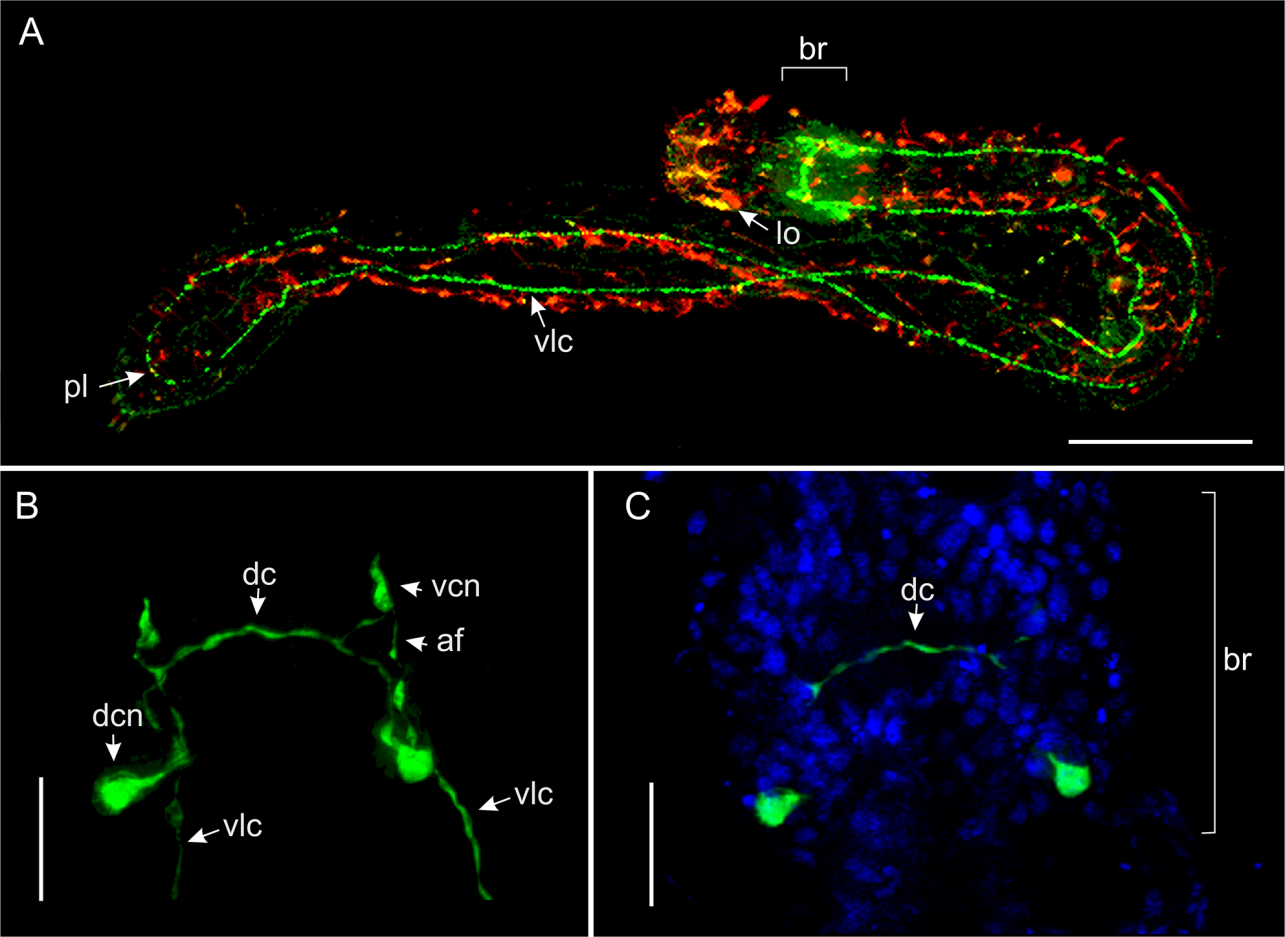
*Thaidasys tongiorgii* gen. et sp. nov. Serotonergic nervous system and nuclear staining visualised with CLSM. A, whole organism. B, serotonergic cerebral ganglion. C, cerebral ganglion stained with DAPI and antiserotonin. af, anterior neurites extended from the dorsal commissure to the ventral somata in the cerebral ganglion; br, cerebral ganglion; dc, dorsal commissure; dcn, dorsal cerebral neurons; lo, leaf-like organ; pl, posterior loop; vlc, ventrolateral cord; vnc, ventral cerebral neurons. Scale bars A = 100 μm, B, C = 10 μm.

**Fig 9:**
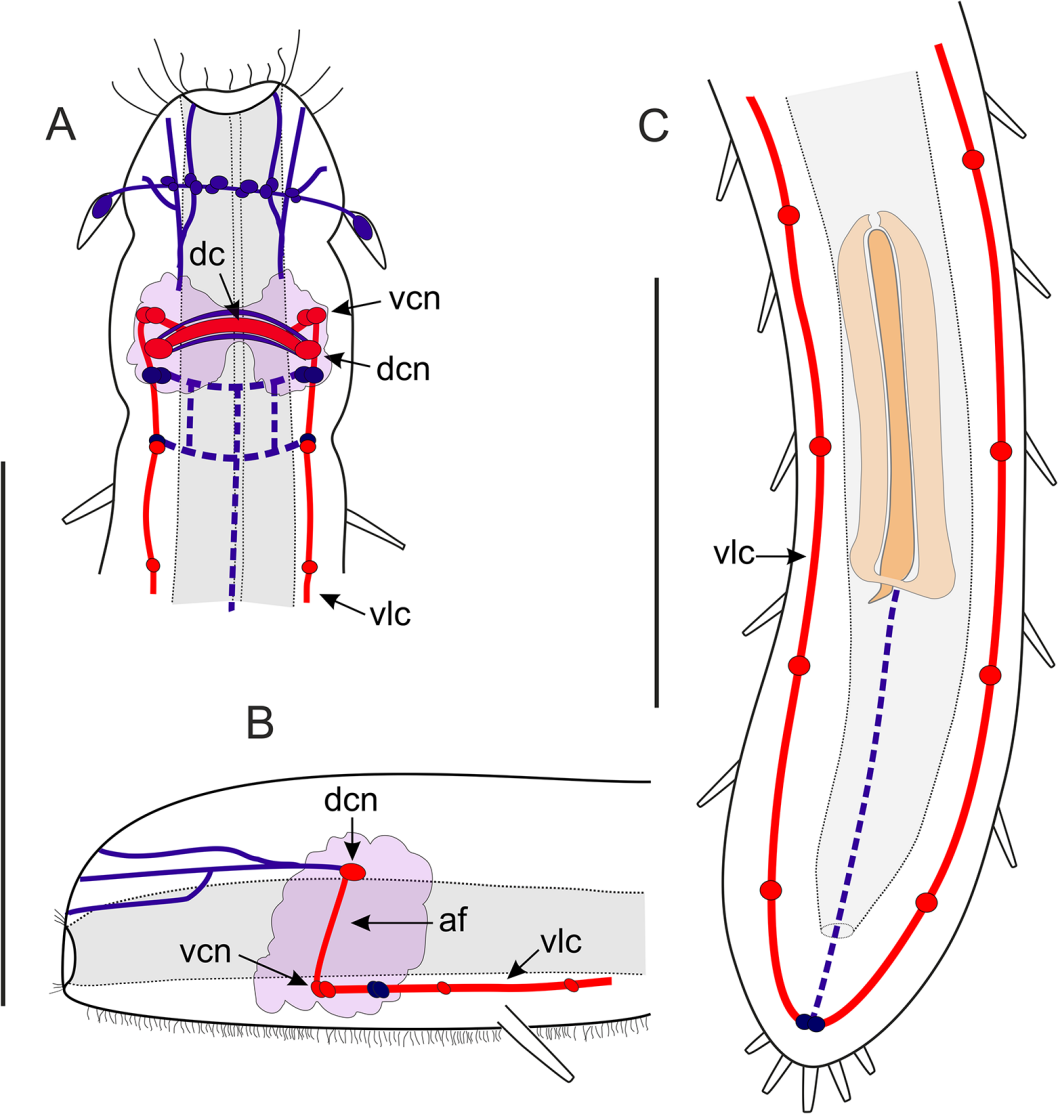
*Thaidasys tongiorgii* gen. et sp. nov. Schematic drawings of the nervous system. The serotoninergic component is emphasised in red. The other components of the nervous system not reactive to serotonin antibodies (e.g., anti-FRMFamide and anti-tubulin) are shown in blue. The anterior neurites connecting the brain to the anterior sensorial cilia are drawn mainly by inference from data on other Macrodasyida. A, anterior region, dorsal view. B, anterior region, lateral view. C, posterior region, dorsal view. af, anterior neurites extended from the dorsal commissure to the ventral somata in the cerebral ganglion; dc, dorsal commissure; dcn, dorsal cerebral neurons; vlc, ventrolateral cord; vcn, ventral cerebral neurons. Scale bars = 100 μm (A, B same scale).

**Fig 10:**
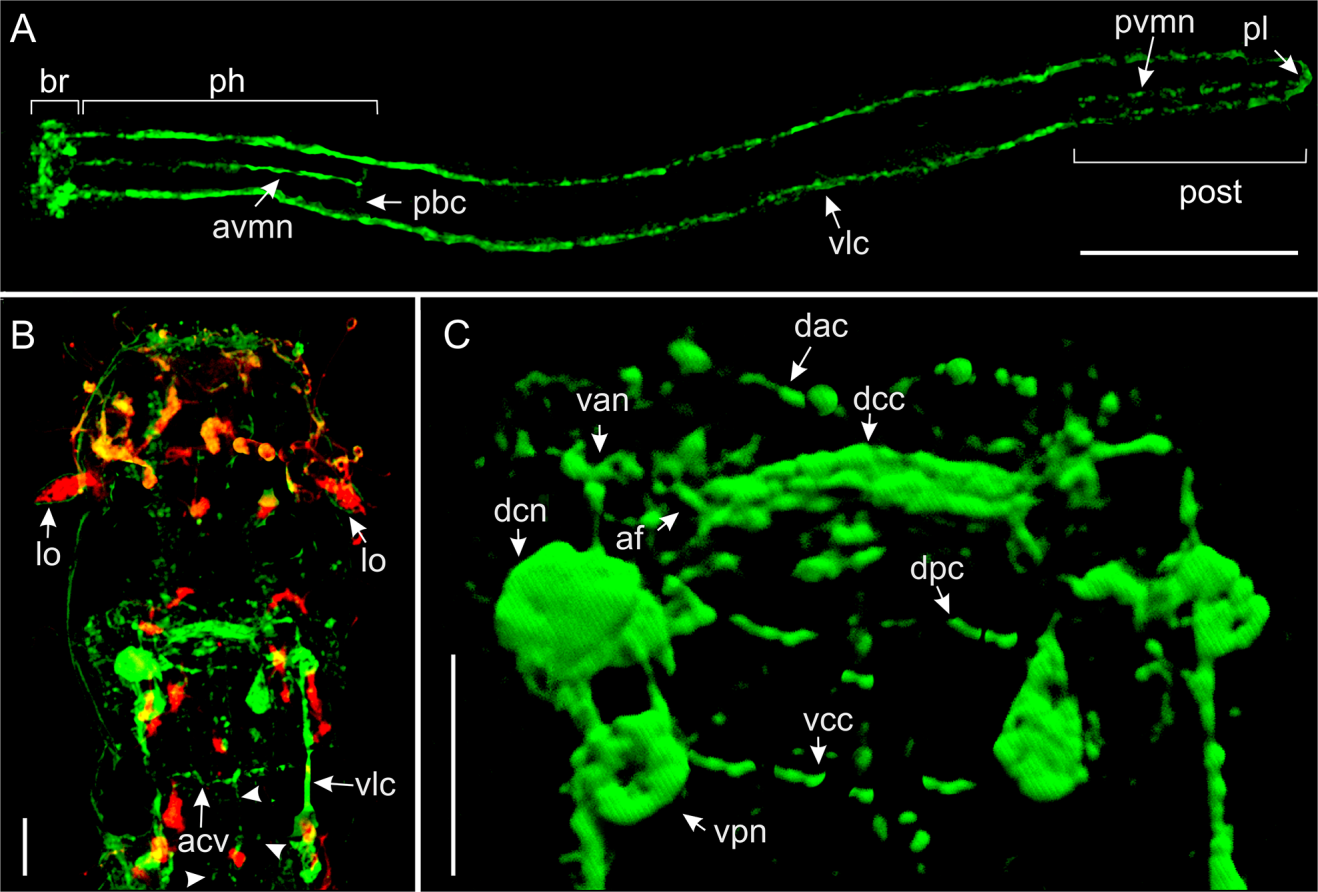
*Thaidasys tongiorgii* gen. et sp. nov. FMRFamide immunoreactive nervous system visualised with CLSM. A, whole organism. B, anterior region, showing the FMRF-IR (green) cerebral ganglion and the anti-tubulin staining (red) in the sensory structures; arrows indicate anterior ventral neurites. C, close up of the cerebral ganglion. acv, anterior commissure of the ventrolateral cords; af, anterior fibres extended from the dorsal commissure to the ventral somata in the cerebral ganglion; avmn, anterior ventromedial neurite; br, cerebral ganglion; dac, cerebral dorsal anterior commissure; dcc, cerebral dorsal central commissure; dcn, cerebral dorsal neurons; dpc, cerebral dorsal posterior commissure; lo, leaf-like organ; ph, pharynx; pbc, ventral commissure at the level of the pharyngo-intestinal junction; pl, posterior loop; post, posterior region; pvmn, posterior ventromedial neurite; van, cerebral ventral anterior neurons; vcc, cerebral ventral commissure; vlc, ventrolateral cord; vpn, cerebral ventral posterior neurons. Scale bars A = 100 μm, B, C = 50 μm.

**Fig 11:**
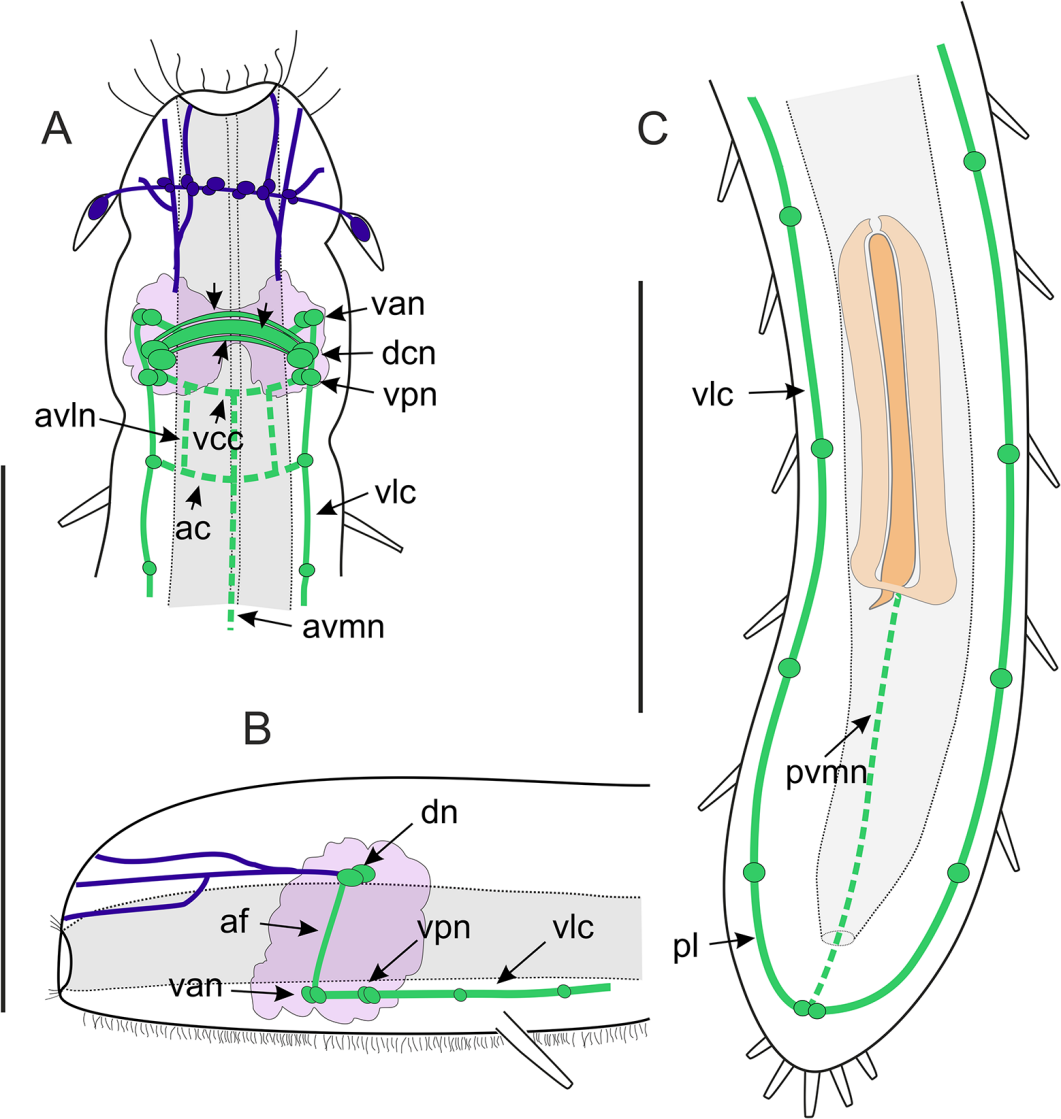
*Thaidasys tongiorgii* gen. et sp. nov. Schematic drawings of the nervous system. The FMRFamide immunoreactive component is emphasised in green. The anterior neurites connecting the brain to the anterior sensorial cilia are drawn mainly by inference from data on other Macrodasyida. A, anterior region dorsal view; arrows indicate the anterior, central and posterior commissures of the cerebral ganglion. B, anterior region, lateral view. C, posterior region, dorsal view. ac, anterior commissure of the ventrolateral cords; af, anterior neurites extended from the dorsal commissure to the ventral somata in the cerebral ganglion; avln, anterior ventrolateral neurite; avmn, anterior ventromedial neurite; dcn, cerebral dorsal neurons; pl, posterior loop; pvmn, posterior ventromedial neurite; van, cerebral ventral anterior neurons; vcc, cerebral ventral commissure; vlc, ventrolateral cord; vpn, cerebral ventral posterior neurons. Scale bars = 100 μm (A, B same scale).

#### Diagnosis

Same as the genus

#### Etymology

The species is named after Paolo Tongiorgi, master and friend, in recognition of his valuable contributions to the field and the endless support to the Italian Gastrotricha research group. The suffix “-*dasys*” is traditionally used in most genera of macrodasyidan gastrotrichs and alludes to their dense ciliation.

#### Examined material

The description of *Thaidasys tongiorgii* gen. et sp. nov. is mainly derived from eight specimens, seven adults and a single juvenile, observed under DIC optics. The holotype, LT = 782 μm, is illustrated in Fig 2A and 2B (International Code of Zoological Nomenclature, Articles 73.1.1, 73.1.4). After observation the physical specimen was fixed in 95% ethanol and later used for DNA analysis (GenBank accession Number KR072683).

The juvenile and the six additional studied adults are no longer extant. Some details about the external morphology are derived from two adults observed with a SEM. Data about musculature and the nervous system came from fifteen (5 + 10) adults observed with CLSM. Three further identified specimens were fixed in alcohol and are kept in the author’s collection together with the SEM prepared specimens. At least fifteen more specimens were examined, in vain, for sperm and/or the frontal organ, and subsequently discarded.

#### Type locality

The sediment sample was collected on 1st February 2010 from the south side of Kata beach, along the South-western coastline of Phuket Island, Thailand (Lat. 07°48'12.35'' N; Long. 98°17'55'' E).

#### Ecology

Numerous in abundance (95% of a sample); intertidal at a water depth of 0.5 m in fine sand (2.34 phi), moderately well sorted (0.94 phi) carbonate sand (kurtosis = 3.43; skewness = -0.64). Values of salinity and temperature of the interstitial water at the time of sampling were 22‰ and 28°C respectively.

#### Description

Based mostly on the adult specimens with a total body length of 782 μm shown in Fig 2. Body elongate and rather narrow, flattened ventrally and vaulted dorsally, with gently undulating sides due to the presence of numerous epidermal glands; cuticular covering smooth, devoid of scales and/or spines (Figs 1A, 1B, 2A, 3D and 5A). Body attaining the maximum width in the mid- to hindgut, and then narrowing again to an ovoidal caudum (Figs 1A, 1B, 2A, 2B and 5A, 5E). Head consisting of two distinct portions. The anterior one, roughly trapezoidal, with rounded angles, appears clearly demarked by a posterior constriction and with a pair of peculiar leaf-like structures, interpreted as sensory organs at U03; it bears sparse sensory cilia on the lateral side, but neither piston pits nor eye spots are present (Figs 1, 2A, 2C, 3B and 4). The posterior portion of the head hosts the brain (see below), it appears slightly inflated and delimited to the rear by a second constriction at U10.

Widths of head\mid-pharyngeal region\mid-trunk\posterior-trunk are as follows: 41\48\64\60 μm at U03\U13\U62\U94, respectively.

Epidermal glands: up to 30 pairs of noticeable epidermal glands irregularly spaced from the posterior end of the head and along the pharyngeal and intestinal regions (from U03 to U94; Figs 1B and 2A); glands are variable in shape, from round to elliptical, and range in size from 9 to14 μm in width and 10–22 μm in length (Fig 2A and 2B); external pores not discernible under DIC optics but visible on the dorsal side under SEM (Fig 4D).

Ciliation: Sensory hairs, up to 22 μm in length, arranged in lateral and dorsolateral columns that are regularly spaced along the body with others, 14–24 μm in length, loosely packed more along the lateral sides of the head. Additional sensory hairs, 15–24 μm in length, forming a dense, semi-circular fringe on the dorsal side of the head (Fig 1).

The ventral locomotory ciliation is in the form of two bands that run separately from under the head (U02) to the posterior end of the trunk (U94). Individual cilia are 12–16 μm in length and appear sparsely packed, especially in the posterior region (Fig 3A).

Adhesive tubes: TbA, 3 per side, 5–8 μm long, forming diagonal columns on each side, and inserting directly on the body surface, just posterior to the oral opening from U01 to U02 (Figs 1C and 3A); TbV, absent; TbVL, 20 per side, 8–10 μm long; six along the pharyngeal region and the remaining along the intestinal region (Fig 1A and 1B); TbD, up to 14 per side, 4–6 μm long, two of which along the posterior half of the pharyngeal region (Fig 1A and 1B); TbDL, absent; TbP, 5–6 in total, 6–10 μm long, surrounding the posterior edge of the caudum (Fig 1A, 1B and 2A).

Digestive tract: Mouth terminal, of medium size (15 μm in diameter, Fig 4A), leading to a short buccal cavity (8 μm in length, Fig 3B); pharynx is 192 μm long, measured from the frontal edge of the head and slightly increasing in width from anterior (15 μm) to posterior (22 μm); pharyngeal pores far from the base at U22, with ventrolateral openings (Figs 1B, 2C and 3C). Pharyngo-intestinal junction at about U26 (Fig 2C). Intestine increases in width from the PhIJ to mid-body and gradually narrows up to the posterior body end; anus ventral at U96 (Fig 1B).

Reproductive tract: Testes and/or sperm absent. Female gonad unpaired, in the second third of the trunk, showing oocytes maturing in a caudo-cephalic direction with largest egg, 128 μm long and 56 μm wide, dorsal to the mid intestine, centered at U62 (Figs 1B, 2A, 2B and 4A). A putative accessory sexual organ in posterior trunk region (U83) is present (Figs 1B, 2A, 2B and 4). The bulky structure is ventral to the intestine, tube-like, up to 76 μm in length and 24 μm in width, with a strongly muscular wall and a canal, 6–8 μm in diameter, running through its length. The canal contains homogeneous, finely-grained material packed in the form of a cigar-like structure whose posterior end appears connected to a sclerotized, hook-like process (Fig 4). In very compressed animals the tip of the sclerotized process protrudes externally from the ventral side. The whole organ is best interpreted as the caudal organ present in other Macrodasyida (see discussion below). Frontal organ absent.

Musculature: The muscular system of *T*. *tongiorgii* gen. et sp. nov. consists of muscles, likely obliquely striated, arranged in internal circular, longitudinal, and helicoidal fibres in the splanchnic compartment, and external somatic longitudinal muscles (Figs 6 and 7). The putative caudal organ (see below) comprises numerous thin fibres spirally arranged (Figs 6C, 6E and 2F). The myoepithelial sucking pharynx is surrounded by numerous, serially arranged visceral muscle rings (1 μm width); outside those are visceral ventral (1–2 μm width) and dorsal (1–2 μm width) longitudinal muscles that stretch along the whole gut tube from the mouth opening to the posterior end (Figs 6A, 6B, 6D, 6E and 7). Two helicoidally arranged muscles insert in the mouth rim (1 μm width; Figs 6B and 7A, 7B) and surround the longitudinal fibres along the pharynx, up to the pharyngo-intestinal junction crossing seven times; no helicoidal muscles are seen in the intestine region (Fig 6A and 6B). Each pharyngeal ventral pore is surrounded by a thin sphincter muscle (1 μm width; Fig 6A). In the intestinal region, posterior to the PhIJ, the visceral longitudinal muscles are surrounded by regularly spaced visceral muscle rings (Fig 7C). Hence, the spatial arrangement of the splanchnic longitudinal and circular muscles is inverted from that of the pharyngeal region that is circular muscles innermost along the pharynx, outermost along the intestine (Fig 7). Thicker somatic ventrolateral paired muscles (2–3 μm width) insert in the mouth rim, extend along the entire body to the rear end, where they join the splanchnic longitudinal muscles (Figs 6A, 6B, 6D, 6E and 7).

Somatic circular muscle (1 μm width) were detected along the intestinal region, especially in the area of the caudal organ; these muscles appeared as serially arranged complete (closed) rings surrounding the somatic longitudinal muscles and all other muscular components (Figs 6E, 6F, and 7C). The caudal organ, ventral to the intestine, shows a series of numerous (at least 70) and thin bands (1 μm width) of spirally arranged muscles enwrapping its canal (Fig 6E and 6F).

Nervous system: Staining of the nervous system of *T*. *tongiorgii* gen. et sp. nov. with DAPI and antibodies against tubulin, serotonin, and FMRFamide revealed a general pattern consisting of 1) a cerebral ganglion involving at least 80 cells, 2) peripheral ventrolateral nerve cords, and 3) anterior sensory structures (Figs 8–11). The cerebral ganglion is centered at about U07; it occupies the posterior region of the head located between the two anterior body constrictions (Figs 8A, 9A, 10B and 11A).

Tubulin immunoreactivity shows the cerebral ganglion as a pair of cluster of dorsal somata connected by a thick commissure. Neurites extend from the commissure in both anterior and posterior directions (Figs 8A, 10B and 11). The posterior neurites extend as the ventrolateral nerve cords that fuse at the posterior end (Figs 8A, 9, 10B and 11). Several perikarya are present along the length of the cords (Figs 8A and 9C).

A strong tubulin-IR signal is also visible at the anterior end in the sensory cilia and in the paired leaf-like organs (lo). Leaf-like organs appeared to be connected by a dorsal commissure, which comprises several perykarya (Figs 10B and 11A, 11B). The anterior sensory structures (cilia and leaf-like organs) are likely linked to the posteriorly located cerebral ganglion by neurites, even though this connection was not visualised in our stainings (Fig 9A and 9B).

Serotonin and FMRFamide immunoreactive structures are generally detectable in the same regions of tubulin, namely in the cerebral ganglion and ventral nerve cords, with some differences (Figs 8–11). Neither serotonin nor the FMRFamide immunoreactive staining was identified anteriorly in the region of the sensory structures. Scattered and inconsistent positive signal along the body was detected at the level of the cuticle, and interpreted as autofluorescence.

The serotonergic nervous system consists of three anterior pairs of somata in the region of the cerebral ganglion. One pair is located dorsally and is connected by fibres to form a dorsal cerebral commissure (26.5 μm length, 1 μm width; Figs 8B, 8C and 9A, 9B). From the dorsal commissure extends two paired longitudinal neurites (Figs 8B and 9A, 9B) that run slightly anteriorly and ventrally until they join two paired serotonin-IR somata, located ventral to the pharynx (Figs 8B and 9A, 9B). These latter neurons extend paired parallel ventrolateral cords (Figs 8A, 8B and 9) that run until joining at the posterior end to form a posterior ventral commissure (Figs 8A and 9C). Several serotonin IR perykarya (1.3–2 μm each) are seen along the ventrolateral cord (e.g. Fig 8B). A weak immunoreactive signal is also detected in a neurite located in the stomatogastric compartment, namely in the central region of the pharynx, from the cerebral ganglion to the pharyngo-intestinal junction (Fig 9A).

FMRFamide immunoreactive staining is present in the cerebral ganglion, along the ventrolateral cords, and in the stomatogastric system (Figs 10 and 11). The cerebral ganglion has paired clusters of 5–7 IR cells (Figs 10C and 11A, 11B) connected by three consecutive cerebral dorsal commissures, the central of which appear broader. (Figs 10C and 11A). The lateral ends of the broad commissure extend neurites to paired ventral, slightly anterior somata (Figs 10C and 11A, 11B), which extend a short posterior neurite to connect additional paired ventral somata (Figs 10C and 11A, 11B). These latter FMRFamide-IR cells are connected with a cerebral ventral commissure and are the origin of the longitudinal ventrolateral nerve cords (Figs 10 and 11). The ventrolateral cords merge in a loop in the rear end and are connected by two FMRFamide-IR peripheral commissures in the anterior region of the body: 1) just past the head, at the level of the first pair of adhesive tubes and, 2) at the base of the pharynx (Figs 10A, 10B and 11A). There are also four very thin FMRF-IR neurites in the splanchnic compartment. Three neurites are visible in the anterior region of the body and one is located in the posterior region. Of the anterior neurites, one is longer and extends medially from the cerebral ventral commissure to the pharyngo-intestinal junction (anterior ventromedial neurite), the other two (anterior ventrolateral neurites) are shorter and extend paired from the cerebral ventral commissure to the first commissure of the peripheral system (Figs 10A, 10B and 11A). The posterior neurite (posterior ventromedial neurite) is visible in between the end of the caudal organ and the ventral posterior commissure (Figs 10A and 11C).

#### Variability and remarks on general morphology

The total body length of the measured adult specimens (i.e. showing a large egg and/or a fully structured accessory sexual organ) ranged from 710 μm to 806 μm (mean = 806 μm ± 33.8 SD, n = 8); maximum body width varied from 55 μm to 68 μm (mean = 61 μm ± 5 SD, n = 8). The number and, to a lesser extent, the arrangement of the adhesive tubes belonging to the different series varied among the observed specimens; in particular, most of the animals showed only two TbA per side whereas the highest number of TbVL, TbD and TbP reached 24, 15 and 6 per side, respectively (for 6 TbD see e.g. Fig 5A). In general, the number of these adhesive tubes was not strictly related to the body length e.g., the longest specimen showed only 2 TbA and 8 TbD per side *vs*. 3 TbA and 15 TbD recorded for a 769 μm specimen. Some differences were noted also with regard to the reproductive structures. More specifically, a 790 μm long specimen showed a full-structured accessory sexual organ but no trace of egg/oocytes; by contrast, a 737 μm long specimen showed a fully mature egg but not the muscular organ. Two subadult specimens, 596 μm and 645 μm in length respectively, showed their putative caudal organ at different stage of development (Fig 4C and 4D) but not the ovary. Finally, the measured juvenile specimen reached 350 μm in total length, with PhIJ at U30; it had 2 TbA, 7 TbVL and 5 TbD per side, and a total of 4 TbP (Fig 3A). None of the reproductive structures were present.

### DNA-based phylogenetic analysis

**Fig 12:**
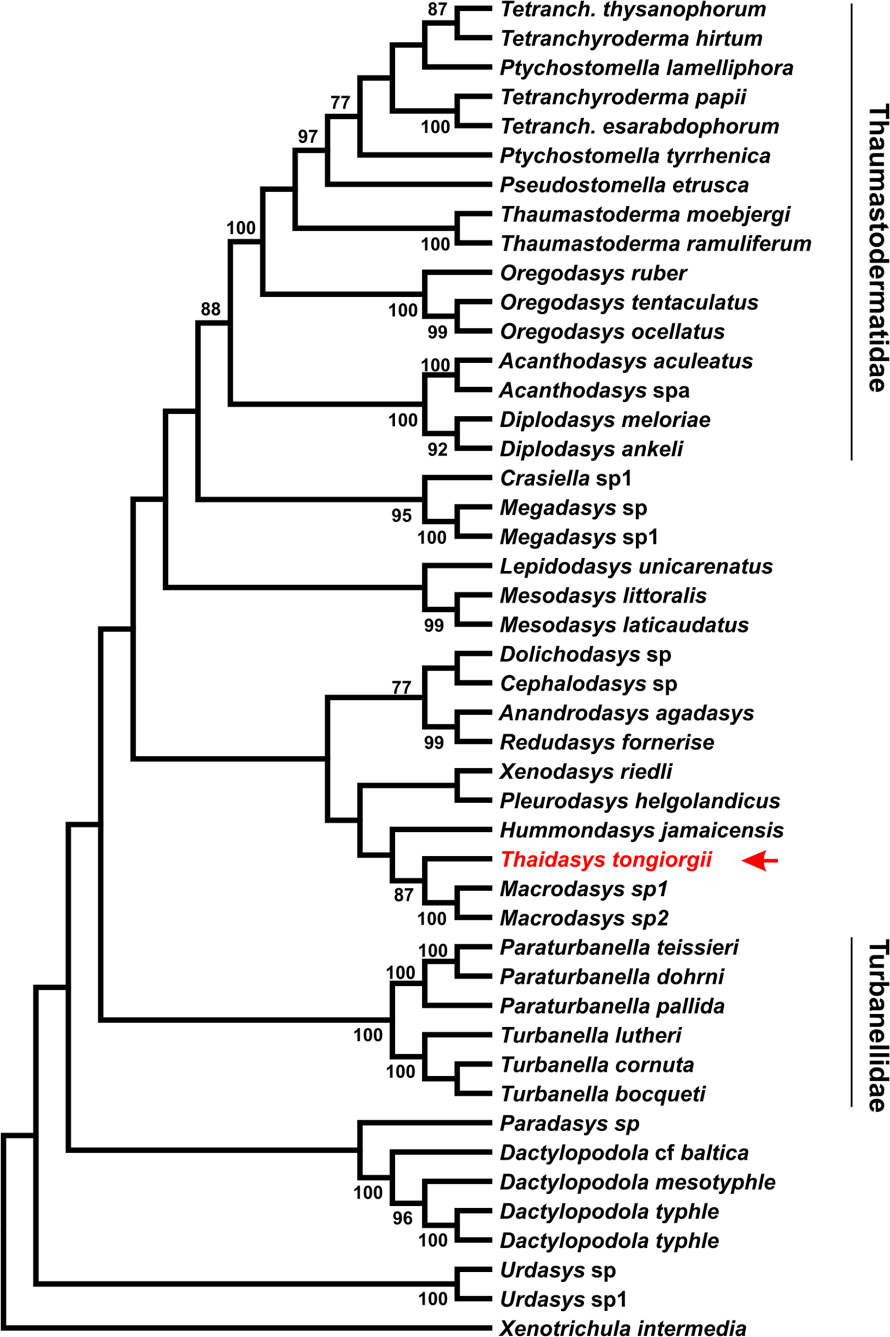
Phylogenetic relationships of *Thaidasys tongiorgii* gen. et sp. nov. inferred from Maximum parsimony analysis of 18S rRNA. The analysis includes 45 Gastrotricha Macrodasyida and the outgroup is represented by *Xenotrichula intermedia* (Chaetonotida, Xenotrichulidae). The most parsimonious tree with length = 3977 is shown. The consistency index is (0.361930), the retention index is (0.580173), and the composite index is 0.232537 (0.209982) for all sites and parsimony-informative sites (in parentheses). Number at nodes represents bootstrap values (1000 replicates).

**Fig 13:**
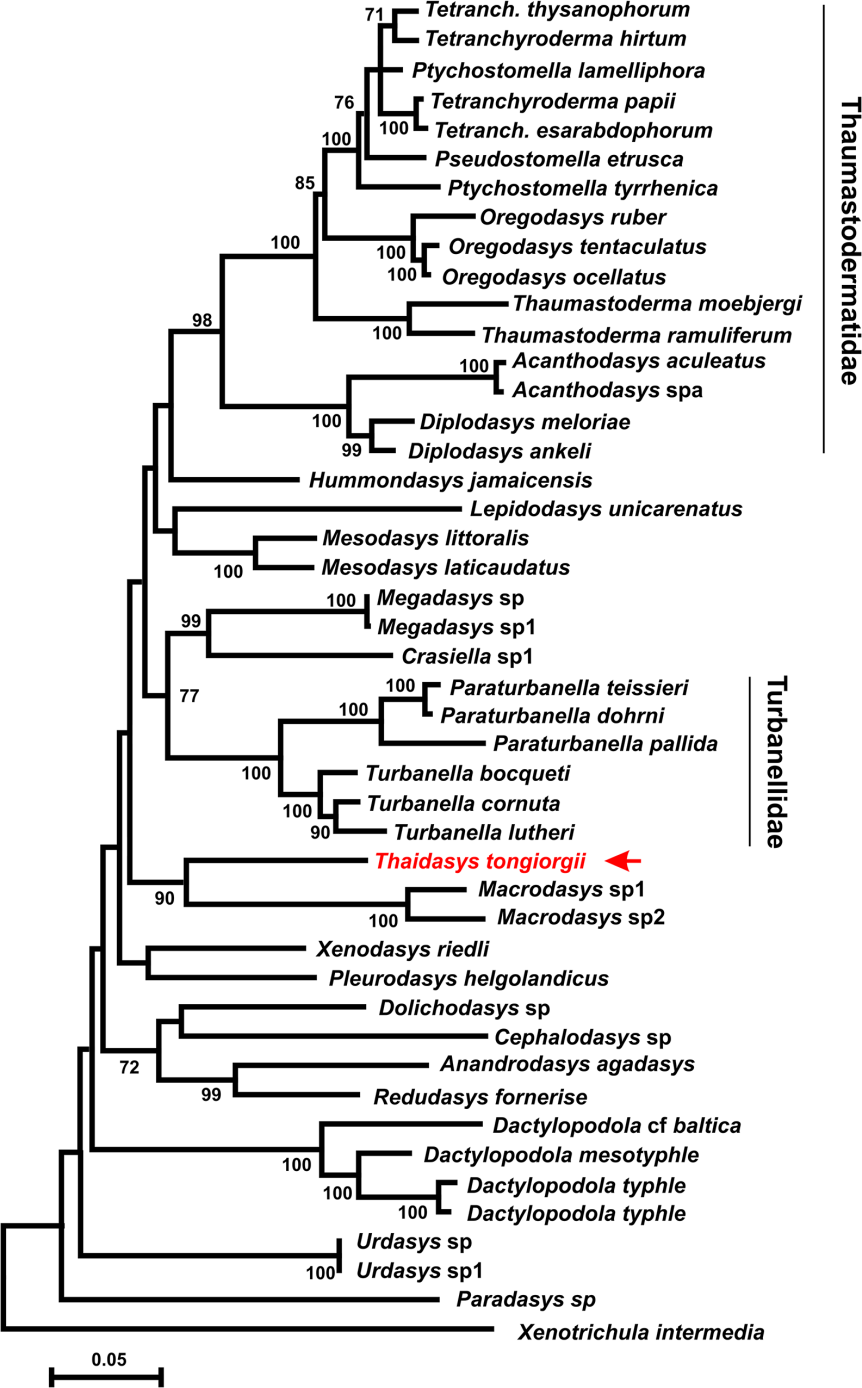
Phylogenetic relationships of *Thaidasys tongiorgii* gen. et sp. nov. inferred from Maximum likelihood analysis of 18S rRNA. The analysis includes 45 Gastrotricha Macrodasyida and the outgroup is represented by *Xenotrichula intermedia* (Chaetonotida, Xenotrichulidae). The tree with the highest log likelihood (-19946.9339) is shown. The tree is drawn to scale, with branch lengths measured in the number of substitutions per site. Number at nodes represents bootstrap values (1000 replicates).

**Fig 14:**
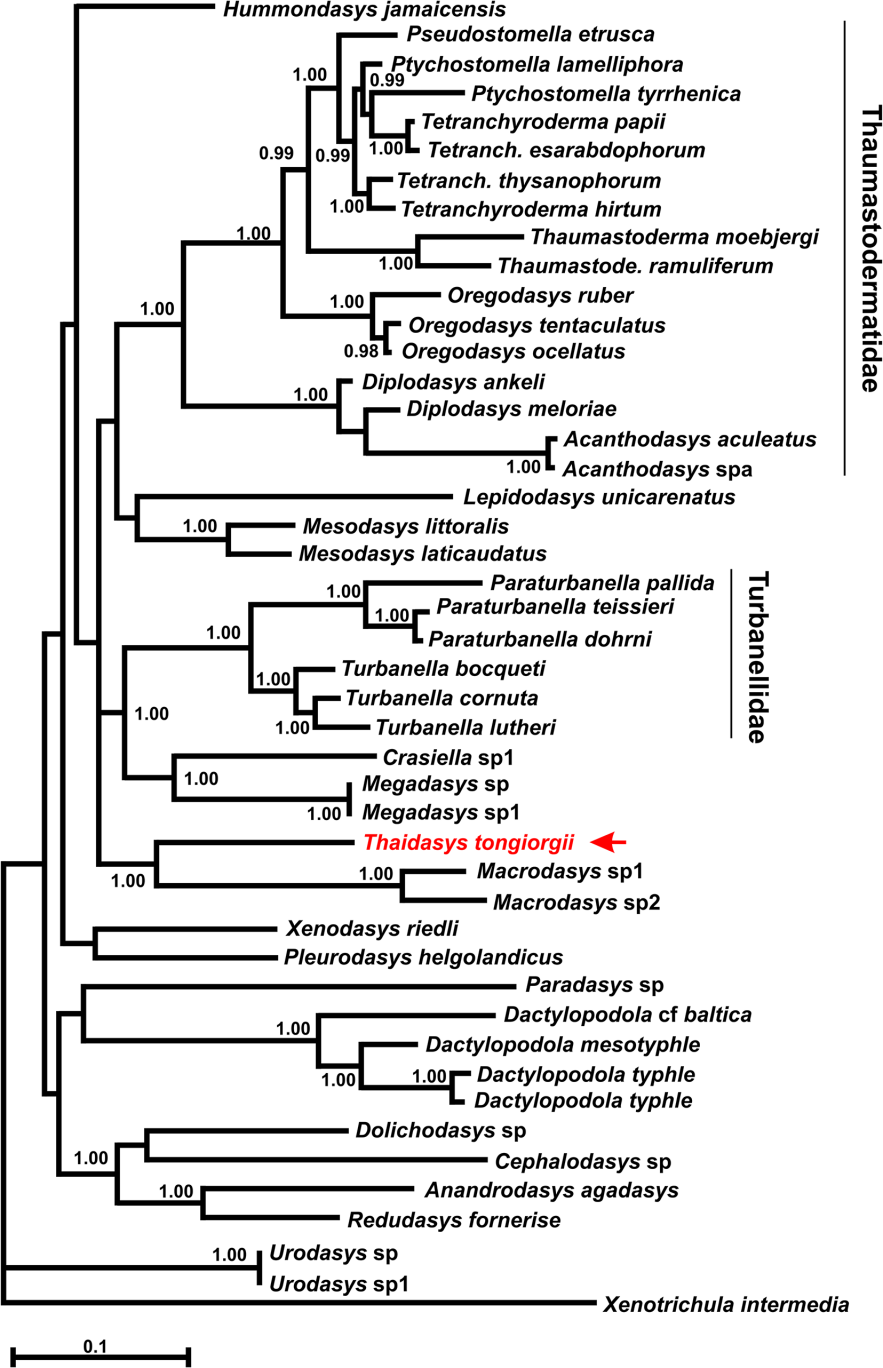
Phylogenetic relationships of *Thaidasys tongiorgii* gen. et sp. nov. inferred from Bayesian analysis of 18S rRNA. The analysis includes 45 Gastrotricha Macrodasyida and the outgroup is represented by *Xenotrichula intermedia* (Chaetonotida, Xenotrichulidae). Number at nodes represents posterior probabilities.

The final rDNA dataset included 1895 alignable positions, 977 of which are constant, and 707 parsimony-informative. The three phylogenetic analyses, carried out with ML, MP and Bayesian approaches, yielded topologies that were congruent with each other, with most of the groups that are in common bearing high nodal support: i.e. bootstrap and Bayesian posterior probability values >70 and 98%, respectively (Figs 12–14). The robustly supported groups include: 1) the densely sampled families Thaumastodermatidae and Turbanellidae and their recognized subgroupings; 2) the recently highlighted alliance between *Redudasys fornerise* Kisielewski, 1987 and *Anandrodasys agadasys* (Hochberg, 2003) (Fam. Redudasyidae, see [24]) and their sister-group relationship with a clade composed of *Cephalodasys* and *Dolichodasys* (Fam. Cephalodasyidae); and 3) the sister-group relationship between *Crasiella* Clausen, 1968 and *Megadasys* Schmidt, 1974, recently united in the family Planodasyidae [33]. In contrast, the currently recognised Macrodasyidae and Cephalodasyidae never appear as monophyletic due to the scattering along the evolutionary tree of their respective species and/or the alliances between members of different families. Genera represented by two or more species were also recovered as monophyletic in the analyses (except *Ptychostomella* Remane, 1926 and *Tetranchyroderma* Remane, 1926). With regard to the new species from Thailand, all of the three analyses strongly indicate (bootstrap ≥ 87, Bayesian posterior probability = 1) a sister-group relationship with the clade containing the two species of *Macrodasys* Remane, 1924 (Fam. Macrodasyidae) involved in the study (Figs 12–14). Finally, the inclusion of the *Thaidasys tongiorgii* gen. et sp. nov., did not produce a consistent find a steady position for the recently described *Hummondasys jamaicensis* Todaro, Leasi & Hochberg, 2014 (Fam. Hummondasyidae, see [21]) in the Macrodasyida phylogenetic tree.

## Discussion

### Diagnostic features and morphology

The metric and meristic information reported for the examined specimens (see above) testify to the variability of some traits (e.g., number and distribution of the adhesive tubes) and highlight the fact that variations are only in part related to the specimen size. With regard to the reproductive system, it seems that the muscular organ begins to form first, at a total length of about 550 μm, while an ovary with distinct oocytes becomes visible when an individual reaches a total length of about 700 μm.

Allowing for the high number of individuals examined, of different sizes/ages, the absence of sperm appears to be the normal condition of *T*. *tongiorgii* gen. et sp. nov. Within Macrodasyida, the absence of spermatozoa was a phenomenon unreported before in specimens provided with accessory reproductive structures. Sperm are absent in parthenogenetic taxa that lack such structures i.e., *Anandrodasys agadasys* Todaro, Dal Zotto, Jondelius, Hochberg, Hummon, Kånneby & Rocha, 2012, *Redudasys fornerise* Kisielewski, 1987 and *Urodasys viviparus* Wilke, 1954.

It could be argued that perhaps the muscular organ of *T*. *tongiorgii* gen. et sp. nov. is not a sexual accessory structure. However, the morpho-functional anatomy of the Gastrotricha, and common sense, suggest otherwise. Therefore, we believe that the massive muscular organ found in *T*. *tongiorgii* gen. and sp. nov. is most likely an accessory reproductive organ because 1) no other structure have been reported in Macrodasyida except for the accessory sexual organs [1], 2) the absence of any alternative hypotheses regarding its function.

The position, in the posterior trunk and ventral to the intestine, shape, and especially the structure (thick muscular wall) lead us to consider the muscular organ found in the new species comparable to the caudal organ present in other Macrodasyida. For instance, a caudal organ similar in many respects to the one present in *T*. *tongiorgii* gen. et sp. nov. has been recently described by Hochberg *et al*. [8] for the Brazilian species *Lepidodasys ligni* Hochberg, Atherton & Gross, 2013. Possible uses of a copulatory organ in animals that lack spermatozoa remains unknown.

The vermiform appearance, along with the cuticular covering made up of only smooth cuticle (i.e., absence of spines and/or scales) and the adhesive tubes of the anterior series (TbA) originating singly and directly from the body surface, make the gastrotrichs from Thailand most similar to some members of the families Cephalodasyidae (i.e., *Dolichodasys* Gagne, 1977 and *Mesodasys* Remane, 1951), Planodasyidae (i.e., *Megadasys* Schmidt, 1974), and to some extent to the recently described *Hummondasys jamaicensis*. In contrast with the new species and members of the taxa mentioned above, *H*. *jamaicensis* possess a posterior body region that is shaped in the form of two caudal pedicles, a trait that makes it clearly distinct from the others [21]. At a level of external anatomy, several differences also emerge between the new species and members of *Dolichodasys*, *Mesodasys* and *Megadasys*, making it difficult to affiliate the Thai species to one of these taxa. *Dolichodasys*, for example, is characterized by a single TbA per side (*vs*. 3) and lateral adhesive structures in form of papillae (*vs*. tubes); *Mesodasys* possesses many TbA regularly arranged over the post oral region (*vs*. 3 tubes per side, arranged in columns) while *Megadasys* possess TbAs that appear as the anterior continuation of the adhesive tubes of the TBVL series and, most importantly, shows a posterior end in the form of a large lobe surrounded by many adhesive tubes arranged in a fan-like fashion (*vs*. 5–6 tubes at the posterior edge shown by the new species).

Clear-cut differences also exist between the new species and taxa mentioned with regard to the reproductive apparatus. These differences go well beyond the simple absence of testicles and sperm in the new species. For instance, *Dolichodasys* shows a single ovary but the eggs develop in an antero-posterior direction, opposite to that in the new species; *Megadasys* possesses a pair of ovaries while the new species has a single ovary. Furthermore, both *Dolichodasys* and *Megadasys* bear two accessory reproductive organs (i.e., frontal- and caudal organ) *vs*. a single organ noted in the new species. *Mesodasys*, similar to the new species, has a single ovary and a single accessory organ; however, the sexual accessory organ present in *Mesodasys* (i.e., caudal organ) is directly connected to the sperm ducts and its wall does not appear to be muscular, which is in contrast with the heavily muscularised wall of the caudal organ present in the new species.

The leaf-like sensorial organs at the posterior lateral sides of the anterior region of the head, and the pharyngeal pores located far off from the pharyngeal base are additional characters that further differentiate the Thai gastrotrichs from all the species belonging to the genera mentioned above, and, when combined, to all the other gastrotrich species known to date.

In conclusion, traits of the external morphology along with the simplified reproductive system call for both the erection of a new species and a new genus for the specimens from Phuket island; consequently, the name *Thaidasys tongiorgii* gen. et sp. nov. is proposed for the new taxon.

Unfortunately, the morpho-functional traits, inclusive of the muscular and nervous systems, of *Thaidasys tongiorgii* gen. et sp. nov. do not permit the formulation of a preferred hypothesis concerning the affiliation of the new genus to any of the currently recognised macrodasyidan families (e.g., [27]).

Guidi *et al*. [33] and Todaro *et al*. [21] highlighted the special relevance that may assume the layout of the reproductive system in the systematisation of the Gastrotricha at a rank of family. In this framework, the simplified reproductive system of the new taxon does not offer robust clues about its potential phylogenetic alliances. In fact, the simplified system of *T*. *tongiorgii* gen. et sp. nov. may be equally interpreted as the result of an evolutionary reduction from one or the other of the different systems found for example in Cephalodasyidae and Planodasyidae, but also in Macrodasyidae, whose members share with the new species the presence of muscular caudal organ (for a review see [1]).

On the other hand, based solely on morphology, it cannot be completely ruled out that such a simplified system may have an alternative origin, including a derivation from an as yet unknown phylogenetic line; a circumstance that could justify the erection of a new family for the new species.

### Muscular and nervous systems

The muscular system of *Thaidasys tongiorgii* gen. et sp. nov. is organised in longitudinal, circular and helicoidal orientation, and it resembles the general arrangement of the musculature so far described for other gastrotrich species (reviewed in [1]).

According to Hochberg [53], the character pattern that was probably present in the last common ancestor of Gastrotricha consists of muscle strands in three different orientations: circular, helicoidal and longitudinal in a splanchnic position, and circular and longitudinal in a somatic compartment. Each arrangement might be present, absent, or differently quantified according to the species. Like other gastrotrichs investigated so far, the new species possesses visceral (= splanchnic) musculature organised as inner circular and external (both ventral and dorsal) longitudinal muscles along the pharynx, and inner longitudinal muscles surrounded by circular rings along the intestine. Helicoidal muscles are present along the pharynx, whereas they are absent posteriorly to the pharyngo-intestinal junction, a condition shared with *Macrodasys caudatus* and *Turbanella ambronensis* [54].

In the somatic compartment, *T*. *tongiorgii* gen. et sp. nov. displays paired longitudinal muscles in a ventrolateral position (*musculus principalis* according to Remane [55]). They insert anteriorly in the mouth rim and run posteriorly past the anus. The insertion in the mouth rim is a condition shared with other taxa that bear TbAs very close to the mouth e.g., *Lepidodasys*, *Macrodasys*, and Thaumastodermatidae. In contrast, species that possess TbAs inserted at some distance from the mouth rim e.g., *Dactylopodola*, *Dolichodasys*, *Paradasys* and and *Turbanella*, possess ventrolateral muscle bands that insert near to where the tubes originate (see [1] for a review).

Somatic circular fibres were seen along the intestinal region but not in the pharyngeal region. As somatic muscles arranged in rings along the entire digestive tube have been described for all macrodasyidans except the Thaumastodermatidae (but see [56]), it is possible that in the new species the somatic circular muscles of the pharyngeal region were not visible due to their reduced thickness and to the strong signal of the pharynx musculature. In Macrodasyida somatic circular muscular along the intestinal region are reported to enclose the ventrolateral muscle bands on either side of the midgut (e.g., [54]). This view has recently been challenged by Kieneke and Schmidt-Rhaesa [1] according to whom the somatic circular muscles surround the somatic longitudinal muscles and probably all the other muscular component. Our finding supports Kieneke and Schmidt-Rhaesa’s hypothesis and call for additional studies to clarify the issue.

*T*. *tongiorgii* gen. et sp. nov. possesses a distinct caudal organ, which is supplied and surrounded by slightly spirally arranged musculature that define an internal canal. Such muscular arrangement in the caudal organ is known to be present in several other species e.g., in *Lepidodasys ligni*, *Macrodasys* sp. and in *Tetranchyroderma papii* [8, 54, 57]. Muscle contractions of the caudal organ are used to support the release of spermatozoa from the caudal organ lumen or, as in *Macrodasys* spp., to evert the copulatory tube (reviewed in [1]). The caudal organ of *T*. *tongiorgii* gen. et sp. nov. appears to be a combination of both the caudal organ of *L*. *ligni* and *Macrodasys* spp. For instance, with the caudal organ of *L*. *ligni*, it shares a lumen that is open at both ends, while like the caudal organ of *Macrodasys* spp. contains a putative copulatory tube (see above).

The nervous system of *T*. *tongiorgii* gen. et sp. nov. resembles what has been described for other gastrotrichs, and namely it consists of a bilateral brain (cerebral ganglion) that encircles the dorsal and lateral anterior regions of the pharynx, and which is connected to a pair of peripheral ventrolateral cords and anterior sensory structures (reviewed in [1] and [58]).

The serotonergic system in macrodasyidan gastrotrichs is known in species of the genera *Dactylopodola*, *Dolichodasys*, *Macrodasys and Turbanella*, [30, 59, 60]. In all species, as in *T*. *tongiorgii* gen. et sp. nov., the serotonergic system is generally present as a pair of cell clusters in the cerebral ganglion connected by a dorsal commissure. These cells project neurites to ventral cells, which project off a paired ventral serotonergic nerve running along the entire body until the posterior end. An exception is *Macrodasys*, which apparently does not show a Serotonin-IR signal in the dorsal commissure [30]. However, as information of *Macrodasys* are based on epifluorescence microscopy, investigation using the more powerful confocal microscopy are needed to confirm the absence of serotonin in the cerebral dorsal commissure of this taxon.

In *T*. *tongiorgii* gen. et sp. nov., a paired serotonin-IR cell was present in the dorsal region of the cerebral ganglion (Fig 9A). These two cells were connected by a dorsal commissure and extended serotonergic fibres to two paired somata located in the ventral side. These ventral cells extended neurites posteriorly to establish a paired ventrolateral serotonin-IR nerve cord, which coalesced posteriorly. In *Dactylopodola baltica*, *D*. *typhle* and *Turbanella cornuta* there are two paired serotonergic cells connected by a commissure in the dorsal region of the cerebral ganglion, whereas *Dolichodasys elongatus*, *Macrodasys caudatus*, *T*. *ambronensis* and *T*. *hyalina* possess, similarly to the new species, only one paired serotonin immunoreactive cell in the same region [30, 60, 61]. Connection of the dorsal somata to ventral perikaria from which neurites extend as the paired ventrolateral nerve cord is a condition that the new species shares solely with *Dactylopodola* [60].

However, as the number of perykaria that are serotonin-IR might 1) depend on the microscopy technique (epifluorescence *vs*. clsm; cfr. [30] *vs*. [60] for *Dactylopodola baltica*), 2) vary across species, and 3) also among individuals of the same species [60], it is difficult to ascertain potential homologies.

*T*. *tongiorgii* gen. et sp. nov. bears the putative ancestral character state of the absence of a ventral serotonin-IR commissure in correspondence of the dorsal one and, as such, it is in particular contrast with *Turbanella* species that do have this ventral commissure [61].

In gastrotricha, the FMRFamide immunoreactivity has, in general, a wider distribution along the nervous system compared to the serotoninergic component. This is also true for *T*. *tongiorgii*, gen. et sp. nov. In the cerebral ganglion of the new species there are three dorsal commissures that are FMRFamide immunoreactive, a condition shared with *Lepidodasys* and *Turbanella* while e.g., *Dactylopodola* and *Xenodasys* possess only two dorsal commissures [60, 62, 63].

The presence of FMRFamide immune reactivity in a commissure ventral to cerebral ganglion is a characteristic common to all gastrotrichs including the new species. On the other hand, *T*. *tongiorgii* gen. et sp. nov. appears unique in that it shows two additional ventral commissures posterior to the brain: one is located just past the head at about U10, and the other at the level of the pharyngo-intestinal junction at U26. It should be emphasized that a second ventral commissure, not too far from the first one, is also present in *Dactylopodola* [60], but it is difficult to say whether it is a homologous condition or not.

The paired nerve cord of *T*. *tongiorgii* gen. et sp. nov. is FMRFamide imunoreactive like all other gastrotrichs investigated so far. However, some differences appear evident. The new species displays a single pair of ventral neurites that project off the brain and run posteriorly the length of the body, whereas *Lepidodasys* and *Turbanella* species possess a pair of additional ventral fibres that project off the brain and reach the anterior end of the body. On the other hand, *Dactylopodola* possesses two paired ventral cords and several anterior projections while *Xenodasys* shows four pairs of nerve cords [60, 63].

In the splanchnic compartment, the new species displays three neurites located in the pharynx and a single neurite that extends ventrally from the end of the genital organ to the posterior end. FMRFamide immune reactivity is widely present in the splanchnic compartment of all macrodasyidans, both in the anterior and posterior body region; however, the general weak and scattered staining might also display different conditions among individuals of the same species, hence, homologies are difficult to determine.

Importantly, we did not perform simultaneous stainings with serotonin and FMRFamide antibodies, so we cannot determine if both neurotransmitters colocalised to the same cell bodies and/or neurites. Still, the new species does show FMRFamide and serotonin in perykarya along the nerve cords.

In short, *T*. *tongiorgii* gen. et sp. nov. shows a immunoreactive to FMRFamide system similar to all gastrotrichs, but with peculiarities unique of this species. Because of the presence of three dorsal commissures, the lower number of neurite projections, the lower number of IR perykarya in the cerebral ganglion, the FMRFamide-IR system of this species resembles more *Lepidodasys and Turbanella* instead of *Dactylopodola* and *Xenodasys* species.

To summarize, both the muscular- and nervous systems of the specimens from Thailand show some peculiarities (e.g., absence of somatic circular muscles along the pharynx, the presence of three serotonin-IR somata in the cerebral ganglion, and a third FMRFamide-IR commissure ventral to the pharyngo-intestinal junction) that may further justify the erection of new genus and species. However, the general arrangement of the muscles and nervous system of the new taxon, similar to that of many other gastrotrichs, makes it difficult to reliably establish potential phylogenetic relationships with any of the other taxa studied so far.

### Phylogenetic remarks

Currently, Gastrotricha systematics is in a state of flux due to the discovery and/or the establishment of new high ranking taxa [21, 23–26] and the conflicts between cladistic studies and the classical systematisation (e.g., [28, 31, 33, 64]). Phylogenetic analysis of the Macrodasyida based on molecular traits (18S rDNA gene alone or in conjunction with the 28S rDNA and Cox 1 genes) have provided support for some of the traditional groupings based on morphological traits but have also unveiled surprising associations, e.g. congeneric species grouping with different families or morphologically disparate species grouping together (e.g., [24, 43, 44]).

Some of the phylogenetic novelties that emerged from molecular analyses have later been confirmed and are considered to be very likely based on re-examination of morphological traits of the taxa using an evolutionary perspective (e.g., [33]). Consequently, it may be said that phylogenetic hypotheses based on molecular markers appear to be credible and, hence, extremely useful in the on-going process of the natural systematisation of the Gastrotricha. This is especially true when the topology of the tree resulting from the analysis appears robust e.g., with high statistical support at nodes and/or results are the same with different computational algorithms. The many statistically supported clades resulting from the current analyses appear, consequently, very robust and likely because 1) concordance among the three analyses, 2) agreement with results from comparable molecular analyses (e.g., [21, 24]) and 3) they are consistent, in general, with the current systematics of the group, which takes into account results from recent morphological investigations [21, 14, 26, 33].

## Conclusion

In our phylogenetic analyses, T. *tongiorgii* gen. et sp. nov. always appears in a sister group relationship with a clade containing the two undetermined species of *Macrodasys* involved in the study; yet, the close phylogenetic alliance of *Thaidasys* and *Macrodasys* is supported by very high statistical values (bootstrap = 87 MP, 90 ML; Bayesian posterior probability = 1; Figs 12–14). Considering the reliability of the evolutionary scenarios based on molecular markers emerged from the previous studies (see above) we consider the position of *Thaidasys* along the Macrodasyida phylogenetic branch suggested by the current study to be highly probable. From a morphological point of view, while there are not evidence against such a scenario, *Thaidasys* and *Macrodasys* share possible synapomorphies defined by the presence of a single ovary and the position of the pharyngeal pores far off the base. Consequently, we propose the affiliation of the new taxon to the Macrodasyidae and offer below an emended diagnosis of the family. In the current analysis, the only other genus affiliated with the Macrodasyidae, *Urodasys*, does not cluster with *Macrodasys* and *Thaidasys*; this presents a conflicting situation that should be addressed in future investigations.

Our knowledge of the biodiversity and phylogeny of the Gastrotricha are far from complete; however, Todaro in Appeltans *et al*. [65] estimated the number of morphological species yet to be discovered to range from 1310 to 1810. The recent find and description of high ranking taxa whose members bear traits that were unreported for Gastrotricha (i.e., *H*. *jamaicensis* and *T*. *tongiorgii*) suggests that among those there may be additional species highly significant from a phylogenetic point of view whose the anatomical peculiarities may shed light on the morphological ground pattern of these creatures and consequently may be helpful for the process of their natural systematisation.

### Emended diagnosis

Macrodasyidae Remane, 1924

Elongate Macrodasyida, up to 806 μm in length (tail excluded); body flattened ventrally and vaulted dorsally covered by naked cuticle. Mouth opening terminal, of medium size. Pharyngeal pores significantly anterior of the pharyngo-intestinal junction. Head indistinct or clearly distinct (*Thaidasys*) from body. Head sensorial structures in form of pestle organs, leaf-like organs (*Thaidasys*) or absent (some *Urodasys*). Posterior end in form of a short tail (*Macrodasys*), long tail (*Urodasys*) or ovoidal (*Thaidasys*). Locomotor ciliation covering the entire ventral side under the head and the pharyngeal region, thereafter forming two longitudinal rows or always forming two separated longitudinal bands (*Thaidasys*). Epidermal glands, inconspicuous or well discernable (*Thaidasys*). Anterior adhesive tubes singly, inserting directly on the cuticle; lateral and/or ventrolateral adhesive tubes present, occasionally numerous (*Thaidasys*); dorsal adhesive tubes absent or present (*Thaidasys*); posterior adhesive tubes on margin of the caudum or along the tail.

Usually hermaphroditic; one species parthenogenetic and viviparous; ovary unpaired or paired (*Urodasys*), egg maturing in caudo-cephalic direction. Testis, paired (*Macrodasys* and some *Urodasys*), unpaired (most *Urodasys*) or absent (*Urodasys viviparus* and *Thaidasys*). Sperm duct(s) opening on the ventral side. Frontal organ, present or absent (some *Urodasys*, *Thaidasys*); caudal organ present as a muscular organ containing a copulatory tube (*Macrodasys* and *Thaidasys*) or a sclerotized stylet (most *Urodasys*), or absent (several *Urodasys*). Intertidal or subtidal in distribution; fine to medium sand, including dysoxic sediments. Type genus:*Macrodasys* Remane, 1924. Other genera: *Urodasys* Remane, 1926 and *Thaidasys* gen. nov.
